# High-Throughput Sequencing and Characterization of the Small RNA Transcriptome Reveal Features of Novel and Conserved MicroRNAs in *Panax ginseng*

**DOI:** 10.1371/journal.pone.0044385

**Published:** 2012-09-04

**Authors:** Bin Wu, Meizhen Wang, Yimian Ma, Lichai Yuan, Shanfa Lu

**Affiliations:** Institute of Medicinal Plant Development, Chinese Academy of Medical Sciences & Peking Union Medical College, Beijing, China; Sun Yat-sen University, China

## Abstract

microRNAs (miRNAs) play vital regulatory roles in many organisms through direct cleavage of transcripts, translational repression, or chromatin modification. Identification of miRNAs has been carried out in various plant species. However, no information is available for miRNAs from *Panax ginseng*, an economically significant medicinal plant species. Using the next generation high-throughput sequencing technology, we obtained 13,326,328 small RNA reads from the roots, stems, leaves and flowers of *P. ginseng*. Analysis of these small RNAs revealed the existence of a large, diverse and highly complicated small RNA population in *P. ginseng*. We identified 73 conserved miRNAs, which could be grouped into 33 families, and 28 non-conserved ones belonging to 9 families. Characterization of *P. ginseng* miRNA precursors revealed many features, such as production of two miRNAs from distinct regions of a precursor, clusters of two precursors in a transcript, and generation of miRNAs from both sense and antisense transcripts. It suggests the complexity of miRNA production in *P. gingseng*. Using a computational approach, we predicted for the conserved and non-conserved miRNA families 99 and 31 target genes, respectively, of which eight were experimentally validated. Among all predicted targets, only about 20% are conserved among various plant species, whereas the others appear to be non-conserved, indicating the diversity of miRNA functions. Consistently, many miRNAs exhibited tissue-specific expression patterns. Moreover, we identified five dehydration- and ten heat-responsive miRNAs and found the existence of a crosstalk among some of the stress-responsive miRNAs. Our results provide the first clue to the elucidation of miRNA functions in *P. ginseng*.

## Introduction

*Panax ginseng* C.A. Meyer, belonging to the genus *Panax* of the family Araliaceae, is an economically significant medicinal plant species and has attracted a lot of attention for the phytomedicinal use since ancient times [1]. Evidence from laboratory and clinical trials showed that the roots of *P. ginseng* could play significant roles in immune system modulation, anti-stress, anti-cancer and anti-diabetes in human beings [2]–[6]. The major bioactive ingredients of ginseng roots are triterpene saponins, which are known as ginsenosides. To date, more than 30 ginsenosides have been identified [7]. Among them, Rb1, Rb2, Rc, Rd, Re, Rf, Rg1 and Rg2 are most relevant to the pharmacological effects of ginseng roots [7]–[9]. In addition to ginsenosides, ginseng roots also contain some other bioactive compounds, such as polysaccharides, phenolics, and flavonoids [10]–[12].

*P. ginseng* is a part shade, low-growing perennial plant native to Asian [7]. It is a tetraploid plant species (2n = 4x = 48) with a large genome. Although the whole genome of *P. ginseng* has not been decoded, the whole chloroplast genome and a large number of expressed sequence tags (ESTs) have been sequenced [13]–[17]. The cDNA and gene sequences currently available includes 31,741 unique sequences assembled from 217,519 high quality 454 sequencing reads [13], 11,412 ESTs obtained from normal cDNA library, and about 563 other cDNA and gene sequences (http://www.ncbi.nlm.nih.gov/). The genetic transformation system of *P. ginseng* has been developed and several ginsenoside biosynthesis-related genes have been characterized through this system. For example, overexpression of the squalene synthase gene *PgSS1* in adventitious roots of transgenic *P. ginseng* resulted in a considerable increase of phytosterol and triterpene saponin (ginsenoside) content [4]. RNA interference (RNAi) of the squalene epoxidase gene *PgSQE1* and the dammarenediol synthase gene *DDS* in transgenic *P. ginseng* led to the reduction of ginsenoside production [18], [19]. It provides great information and tools for studying gene functions and regulatory networks in *P. ginseng*.

microRNAs (miRNAs) are a class of small endogenous non-coding RNAs. They originate from long primary transcripts (pri-miRNAs), which have stem-loop structures [20]. In plants, miRNAs regulate target genes either through direct cleavage of transcripts [21], [22] or translational repression at post-transcriptional level [23], or, in some case, by methylation at transcriptional level [24]. It has been shown that miRNAs play important regulatory roles in plant development and stress responses [25]–[30]. For example, miR172 regulates the development of flowers by targeting APETALA2 (AP2) and its homologs with two tandem AP2 DNA-binding domains [31]–[33]. miR160 is involved in root development through the regulation of auxin response factors (ARFs) [32]–[34]. An increasing number of stress-responsive miRNAs are being identified [28], [30], [35].

Since the first discovery of plant miRNAs from *Arabidopsis*, plant miRNAs have been intensely studied using experimental and computational approaches [36], [37]. The number of miRNAs identified has reached to 4011 according to miRBase release 18 [38]. However, there is no information available for miRNAs from *P. ginseng*. In this study, we constructed a small RNA library of *P. ginseng* and carried out high-throughput Illumina sequencing. We identified a total of 101 conserved and non-conserved miRNAs from the small RNA dataset. Computational analysis of miRNAs and their precursor sequences revealed some features of *P. ginseng* miRNAs. Many non-conserved *P. ginseng* miRNAs showed tissue-specific expression and some of them responded to dehydration and heat treatments. Consistently, many predicted targets of non-conserved *P. ginseng* miRNAs are associated with metabolism, signal transduction and stress responses.

## Results

### Features of *P. ginseng* Small RNA Population

In order to identify small RNAs in *P. ginseng*, we generated a small RNA library using pooled RNA isolated from roots, stems, leaves and flowers. The library was then sequenced by the high-throughput Illumina sequencing technology [39]. A total of 13,326,328 raw sequence reads were obtained. After removing adaptors, low quality sequences and those smaller than 18 nt, 12,000,591 clean reads with sizes ranged from 18 to 30 nt were collected (Table S1). They were represented by 1,502,664 unique small RNA sequences, of which 114 were sequenced more than 10,000 times, whereas 1,178,394 (78.4%) and 152,436 (10.1%) were sequenced only one and two times, respectively. It indicates that a large, diverse and highly complicated small RNA population exists in *P. ginseng*. Additionally, 30,240 small RNAs, representing 1,283,459 reads, could be mapped to the chloroplast genome of *P. ginseng* using the SOAP2 program with no mismatches allowed [16], [40], indicating some small RNAs are probably originated from the chloroplast genome.

**Figure 1:**
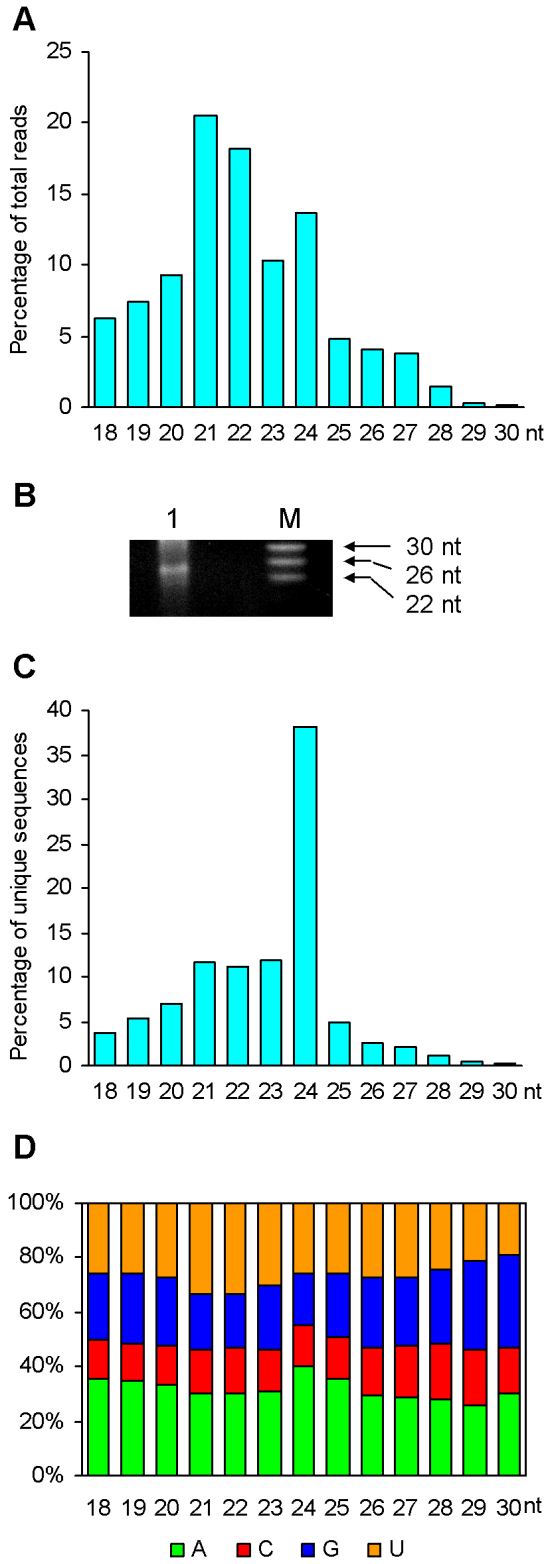
Analysis of *P. ginseng* small RNA population. (A) Size distribution of clean reads. (B) Electrophoretic analysis of small RNAs. Low molecular weight (LMW) RNA was resolved on a 15% denaturing polyacrylamide gel and stained with ethidium bromide. The sizes of RNA ladder are indicated. (C) Size distribution of unique small RNA sequences. (D) Percentage of unique sequences starting with an A, C, G, and U in each size group.

Among 12,000,591 clean reads, the majority (71.22%) range from 20 to 24 nt in length, wherein the 21 (20.24%) and 22 (18.03%) nt ones are two major size groups (Figure 1A). The group of 24 nt clean reads also accounts for a relatively high proportion (13.54%), whereas it is significantly less compared with those identified from *Arabidopsis* [41], *Medicago truncatula* [42], *Brachypodium distachyon* [43], *Arachis hypogaea* [44], *Citrus trifoliate* [45], *Gossypium hirsutum* [46], *Oryza sativa* [47] and *Zea may* [48], where the 24 nt group is the most abundant. Therefore, we analyzed the pattern of low molecular weight (LMW) RNA from the pooled total RNA using gel electrophoresis as previously described [49]. Unexpectedly, the results of electrophoresis show that the most abundant group of small RNAs is 24 nt (Figure 1B). The inconsistency of results from electrophoresis and high-throughput sequencing was also observed in grape, in which a nearly equal number of 21 nt and 24 nt small RNAs was detected by electrophoresis, whereas the number of 21 nt sequence reads are more than five times of the 24 nt reads [50]. The factors causing the inconsistency remain to be elucidated.

Size distribution analysis of the 1,502,664 unique small RNA sequences showed that the group of 24 nt small RNAs was the biggest, which accounted for 38.24% of total unique sequences (Figure 1C). It suggests that 24 nt small RNAs are the most diverse in *P. ginseng*, although their average of abundance is less compared with those in many other plant species. The groups of 21 and 22 nt have the highest percentage of small RNAs beginning with a U nucleotide, whereas the 24 nt group enriches with sequences having an A at the 5′ end (Figure 1D). It indicates a great proportion of miRNAs and *trans*-acting small interfering RNAs (ta-siRNAs) in the 21 and 22 nt groups and significant amount of repeat-associated siRNAs (ra-siRNAs) and natural antisense transcript-derived siRNAs (nat-siRNAs) in the 24 nt group, since miRNAs and ta-siRNAs tend to begin with a U and ra-siRNAs and nat-siRNAs prefer an A at the 5′ end [51]–[55]. It is consistent with features of small RNAs from other plant species, such as *Arabidopsis* and *Populus* [54], [56]. The diversity of 24 nt ginseng small RNAs may indicate the complexity of *P. ginseng* genome, although the whole genome has not been decoded.

### Identification of Conserved miRNAs

Using the SOAP2 program, we mapped 1727 of 1,502,664 unique small RNA sequences to known miRNA precursors with no more than 2 mismatches [38], [40]. Among them, 661 and 41 unique sequences, representing 305,744 and 406 reads, were mapped to mature miRNA and miRNA* regions, respectively, whereas the other 1025 sequences, representing 126,128 reads, were mapped to other regions of known miRNA precursors. Further alignment of the small RNAs mapped to miRNA regions of known precursors allowed us to group the 661 sequences into 73 miRNA candidates, members of 33 conserved miRNA families (Table S2). Similarly, the 41 unique sequences mapped to miRNA* regions were grouped into 22 miRNAs* putatively produced from 9 gene families, including *MIR156*, *MIR166*, *MIR167*, *MIR171*, *MIR172*, *MIR390*, *MIR396*, *MIR482*, and *MIR4376* (Table S3).

Among the 33 identified miRNA families, 22 are deeply conserved among many plant species, whereas the other 11 have less evolutionary conservation. For instance, *MIR156*, *MIR166*, *MIR169*, *MIR319* and *MIR394* have been identified from more than 40 monocots and dicots, whereas *MIR403* is found only in several dicots, such as *Arabidopsis*, *P. trichocarpa*, *Vitis vinifera*, and *Ricinus communis* [30], [38], [57]–[59]. The other 10 miRNA families with less conservation include *MIR1439*, *MIR5072* and *MIR5079* that were previously identified from rice only, *MIR1509* and *MIR1510* found in soybean and *Medicago*, *MIR894* and *MIR1024* identified from *Physcomitrella patens*, *MIR1863* found in rice and Norway spruce, *MIR4376* found in soybean and tomato, and *MIR5139* previously identified from a medicinal plant species, *Rehmannia glutinosa* [42], [60]–[72]. The identification of less conserved miRNAs indicates the specificity and complexity of *P. ginseng* miRNAs.

**Figure 2:**
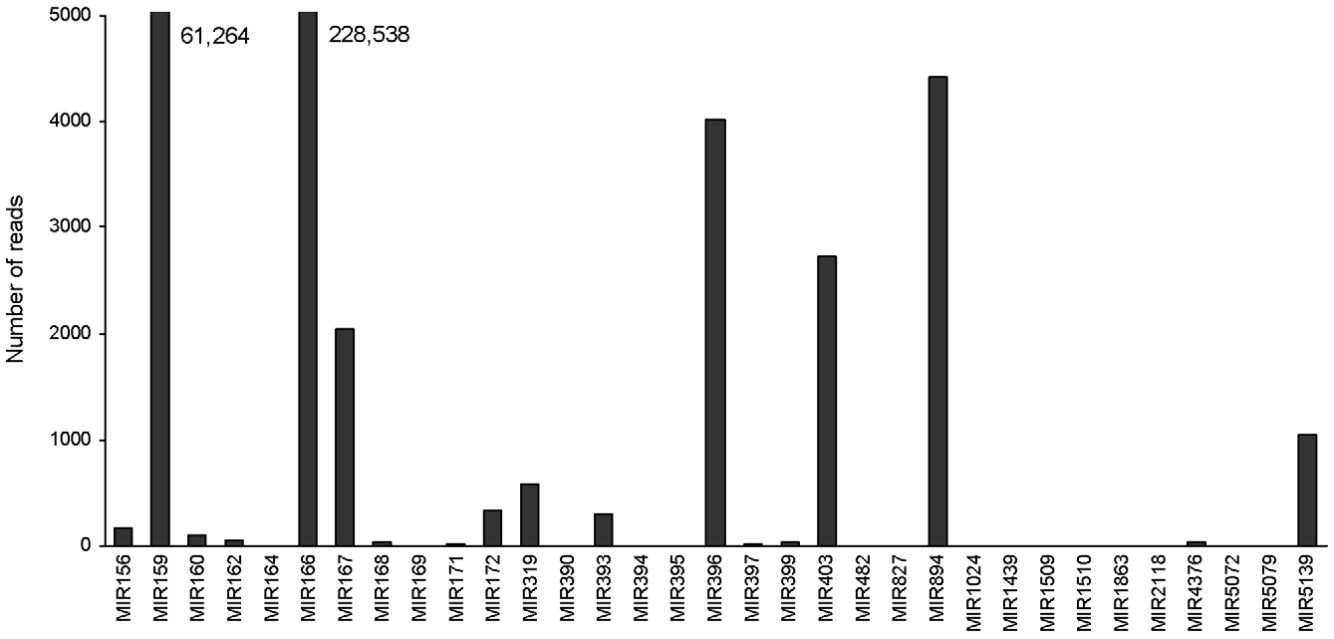
Number of reads for the conserved miRNA families. The read numbers of MIR159 and MIR166 are shown.

It has been suggested that the expression profiles of miRNA genes can be estimated by high throughput sequencing-based technology [73], [74]. Among the 33 conserved miRNA families, *MIR166* was sequenced 228,538 times, which accounted for 74.8% of total conserved miRNA reads, suggesting it is the most abundant miRNA family in the *P. ginseng* tissues analyzed (Figure 2). The second abundant miRNA family is *MIR159*, which was sequenced 61,264 times and accounted for 20.0% of total conserved miRNA reads. The other conserved miRNA families are less abundant and each has less than 1.5% of total conserved miRNA reads. Among them, 15 have less than 10 reads in our small RNA data set (Table S2). Compared with other plants, *P. ginseng* has significantly different expression profiles for some miRNAs. For example, *MIR156* is highly expressed in peanut [44], whereas its expression in *P. ginseng* is low (Figure 2). These results suggest the differential expression of miRNAs in *P. ginseng* and indicate the specificity of miRNA expression in different plant species.

We mapped the 73 conserved miRNAs to unigenes of *P. ginseng* using the SOAP2 program and then analyzed secondary structures of the unigenes using the mfold program [40], [75]. As a result, four miRNA precursor sequences were identified. Two of them are precursors of *MIR482*. The other two generate miR2118 and miR4376 (Figure S1). Further mapping of the small RNAs to miRNA precursors allowed the identification of three additional miRNA* sequences: one for miR482a, one for miR4376, and the other for both miR482b and miR2118 (Table S3). No precursor sequences for other conserved miRNAs were identified. It could be due to the limited number of *P. ginseng* unigenes.

### Identification of Non-conserved miRNAs

In order to identify non-conserved miRNAs as much as possible, we used two computational approaches. First, we mapped small RNAs to 31,371 unigenes of *P. ginseng* using the SOAP2 program with no mismatches allowed [40] and analyzed the secondary structures of unigenes with small RNA mapped using the mfold program [75]. Next, we used MIREAP software (http://sourceforge.net/projects/mireap/) to predict precursors from *P. ginseng* unigenes. In total, we identified 25 miRNA precursors. These precursors produced a total of 28 mature miRNAs. We then submitted the precursors and the corresponding mature miRNAs to miRBase for official names [38]]. Based on sequence similarities, the 25 miRNA precursors were grouped into 9 miRNA gene families, although some members of a family generated distinct mature miRNA sequences (Table 1, Figure S1). Except *MIR6135* and *MIR6140* families, which have 11 and 4 members, respectively, the other 7 non-conserved miRNA gene families have only one or two members. The results showing few members for non-conserved miRNA families have been previously found in other plant species, such as *Arabidopsis* [76]. In addition to miRNAs, we identified a miRNA* sequence for the non-conserved miRNA, miR6136b, which verified computational prediction of non-conserved miRNAs (Table 1).

**Table 1:** Non-conserved miRNAs and miRNAs* in *Panax ginseng*.

miRNA name	miRNA sequence (5′→3′)	Size (nt)	reads	MFEI^a^		Unigene ID		Length (bp)		
miR6135a	TGGTAAGTTGGTCAATTGGC	20	107		1.49		PUT-183a-Panax_ginseng-10400		390	
miR6135b	TGGTAAGTTGGTCAATTGGC	20	107		1.48		PUT-183a-Panax_ginseng-1888		3603	
miR6135c	GGGTAAGTTGGTCAATTGAC	20	17		1.45		PUT-183a-Panax_ginseng-1888		3603	
miR6135d	GGGTAAGTTGGTCAATTGAC	20	17		1.66		PUT-183a-Panax_ginseng-5770		779	
miR6135e.1	GGGTAAGTTGGTCAATTGAC	20	17		1.47		PUT-183a-Panax_ginseng-7458		799	
miR6135e.2	AATTGACTAATAGAATACTGACAC	24	21		1.47		PUT-183a-Panax_ginseng-7458		799	
miR6135f	GAGTAAGTTGGTCAATTGGC	20	4		1.32		PUT-183a-Panax_ginseng-1153		514	
miR6135g	GAGTAAGTTGGTCAATTGGC	20	4		1.63		PUT-183a-Panax_ginseng-11661		155	
miR6135h	GTAAGTTGGTCAATTGGC	18	5		1.02		PUT-183a-Panax_ginseng-17044		767	
miR6135i	AATTGGCCAATAGAATACTGACAC	24	30		1.43		PUT-183a-Panax_ginseng-5770		779	
miR6135j	AATTGACTAATAGAATACTGACAC	24	21		1.32		FW1NBNE01BCPRY		474	
miR6135k	CGTGTCGATACTGTATTGGT	20	3		1.54		PUT-183a-Panax_ginseng-1587		475	
miR6136a.1	TAGACGACGGTTGTATGACCG	21	32		1.40		FW1NBNE01BEGNB		465	
miR6136a.2	ACGGGTGAGTAAGATAAGGGGTAT	24	88		1.40		FW1NBNE01BEGNB		465	
miR6136b	TCATACAACCGTCGTCTATAC	21	70		1.45		FW1NBNE01BEGNB		465	
miR6136b*	TATAAATGATGGTTGTATGAC	21	1		1.45		FW1NBNE01BEGNB		465	
miR6137a	ATGAAAATTGTCGCTATAGATC	22	3		2.13		FW1NBNE01A9PXQ		425	
miR6137b	ATGAAAATTGTCGCTATAGATC	22	3		1.56		PUT-183a-Panax_ginseng-4023		606	
miR6138	TACGTTTGGATTGAAGGAATGAAA	24	13		0.98		PUT-183a-Panax_ginseng-10763		517	
miR6139	AAGAATCATTGGGAAGGGAAGAAA	24	9		1.15		FW1NBNE01AHR0J		608	
miR6140a	AATGTTTGTAGAATAGTTTGTGTC	24	3		1.38		FW1NBNE01A9GRG		391	
miR6140b	AATGTTTGTAGAATAATTTGTGTA	24	2		1.14		PUT-183a-Panax_ginseng-19298		1417	
miR6140c	GCTGAGGTGGAGTATGCCACATC	23	5		1.32		FW1NBNE01ATMKX		436	
miR6140d	CGTTGATGTGGCATACTTCACC	22	3		1.12		PUT-183a-Panax_ginseng-8684		972	
miR6141	TAACTAAATCTGGCCTGTAGCGGA	24	7		1.56		FW1NBNE01B00A0		391	
miR6142	GACGATTTTTTGGGCTATGACGAC	24	5		0.93		FW1NBNE01CDN8L		235	
miR6143a	AGTACTGTATTGGGCATGAAG	21	13		1.20		FW1NBNE01CELC5		535	
miR6143b-5p	ACAATGTCGACACGCAGGCGGAGA	24	2		1.24		PUT-183a-Panax_ginseng-16660		399	
miR6143b-3p	CAGCACTGTATTGAACATGAA	21	4		1.24		PUT-183a-Panax_ginseng-16660		399	

a MFEI, minimal folding free energy index of the hairpin structures.

### Special Features of miRNA Genes in *P. ginseng*

**Figure 3:**
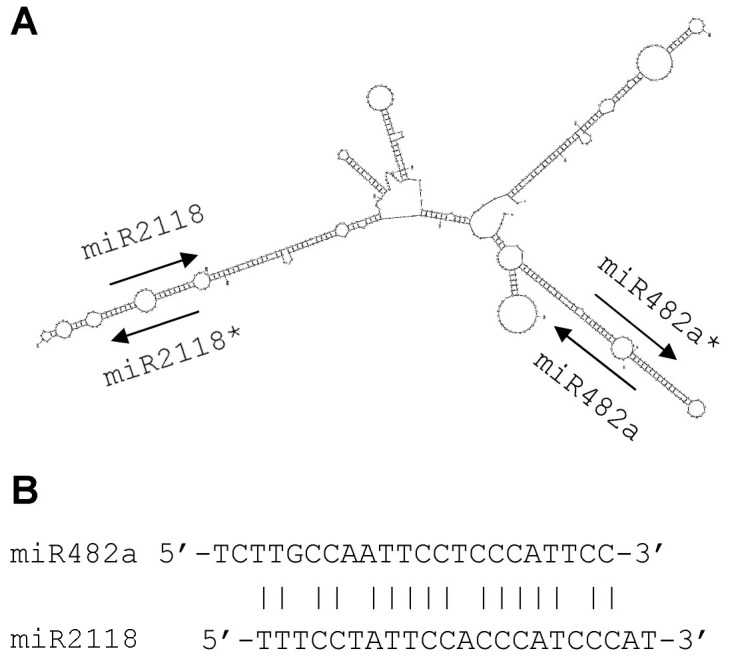
The MIR482a and MIR2118 precursors cluster in a unigene. (A) The secondary structure of PUT-183a-Panax_Ginseng-17125 was predicted by the mfold program using the default parameters [75]. Arrows indicate the position and orientation of miRNAs and miRNA*s. (B) Alignment of the mature miR482a and miR2118 sequences. Vertical dashes indicate the Watson-Crick pairing between miR482a and miR2118 sequences.

The precursors of miR482a and miR2118 cluster in a unigene, PUT-183a-Panax_ginseng-17125 (Figure 3A). Existence of two precursors is verified by the finding of miR482a* and miR2118* in our small RNA data set. Clustering of miRNAs is a universal phenomenon in animal [77]–[79]. It has also been observed for some plant miRNAs, such as some members of the *MIR156* family in wheat and tobacco [80], [81], some members of the *MIR166* family in *Arabidopsis*, rice, wheat and soybean [21], [81], [82], moss miR1219a and miR1219b [83], some member of the *MIR395* family in *Arabidopsis* and wheat [41], [81], switchgrass miR2118a and miR2118b [84], loblolly pine miR950a and miR950b [85], and *Pinus pinaster* miR1314a and miR1314b [73]. Both miR482a and miR2118 are located in the 3′ arm of hairpin structures and are unidirectional in the unigene (Figure 3A). The presence of two miRNA precursors in a unigene indicates that the generated miRNAs are co-regulated and are probably involved in the network of a cellular process [77]–[79]. Consistently, miR482a and miR2118 show a high sequence similarity (Figure 3B), which enable them to regulate same targets and/or different members of a gene family in *P. ginseng*.

Several miRNA precursors generate small RNAs from distinct regions (Figure S1). For instance, both miR6136a.1 and miR6136a.2 are derived from FW1NBNE01BEGNB. PUT-183a-Panax_ginseng-7458 produces two putative miRNAs, including miR6135e.1 and miR6135e.2. Similarly, PUT-183a-Panax_ginseng-16660 generates two mature miRNAs, miR6143b-5p and miR6143b-3p. miR6135e.1 and miR6135e.2 are located in the 5′ arm of a hairpin structure, miR6136a.1 and miR6136a.2 are located in the 3′ arm, whereas miR6143b-5p and miR6143b-3p are produced from different arms of a hairpin structure. Generation of multiple miRNAs has previously been observed for several conserved miRNA genes, such as *Arabidopsis MIR159*, *MIR319*, *MIR163*, *MIR822*, *MIR829* and *MIR839*, rice *MIR159* and *MIR319* [50], [52], [86], [87]. Both MIR159 and MIR319 precursors generate multiple miRNAs in a phased loop-to-base manner [88], [89]. However, mechanisms for the production of multiple miRNAs from a precursor remain to be elucidated in *P. ginseng*.

Three unigenes generating non-conserved miRNAs could be bidirectionally transcribed (Table 1, Figure S1). It includes PUT-183a-Panax_ginseng-1888, PUT-183a-Panax_ginseng-5770, and FW1NBNE01BEGNB. PUT-183a-Panax_ginseng-1888 produced miR6135b, whereas its antisense transcript was predicted to generate miR6135c. Similarly, PUT-183a-Panax_ginseng-5770 and FW1NBNE01BEGNB are precursors of miR6135d and two members of *MIR6136* family (miR6136a.1 and miR6136a.2), whereas their antisense transcripts produce miR6135i and miR6136b, respectively. Bidirectionally transcribed miRNA precursors were previously reported in various animals and plants, such as *Drosophila* [90]–[92], soybean [82], and switchgrass [84]. Because the transcription direction of these ginseng unigenes is unknown, it is possible that one or both of the predicted precursors could be authentic.

### Conserved and Non-conserved Targets of Conserved miRNAs

Plant miRNAs show perfect or near-perfect complementarities to their targets [93], [94]. It allows an effective prediction of miRNA targets through computation. To predict the targets of miRNAs in *P. ginseng*, we used psRNATarget, a useful web server, which searches potential targets using an improved iterative parallel Smith-Waterman algorithm and a weighted scoring schema and calculates penalty scores for mismatched patterns in the miRNA:mRNA duplexes within a 20-base sequence window [95], [96]. With the application of penalty score cutoff threshold 0–3, we identified a total of 99 unigenes from 31,371 assembled *P. ginseng* unigenes to be targets of 28 conserved miRNA families (Table S4). The number of targets for each miRNA family ranges from one to eight. Each unigene is predicted to be the target of a conserved miRNA except the TIR-NBS disease resistance protein gene, FW1NBNE01A3PPX, which is targeted by pgi-miR482 and pgi-miR1510. No targets were found for *MIR160*, *MIR168*, *MIR319*, *MIR894*, and *MIR1024* in the unigene set. It could be due to the limited number of *P. ginseng* unigenes or the low expression levels of their targets in the *P. ginseng* materials used for EST library construction.

**Figure 4:**
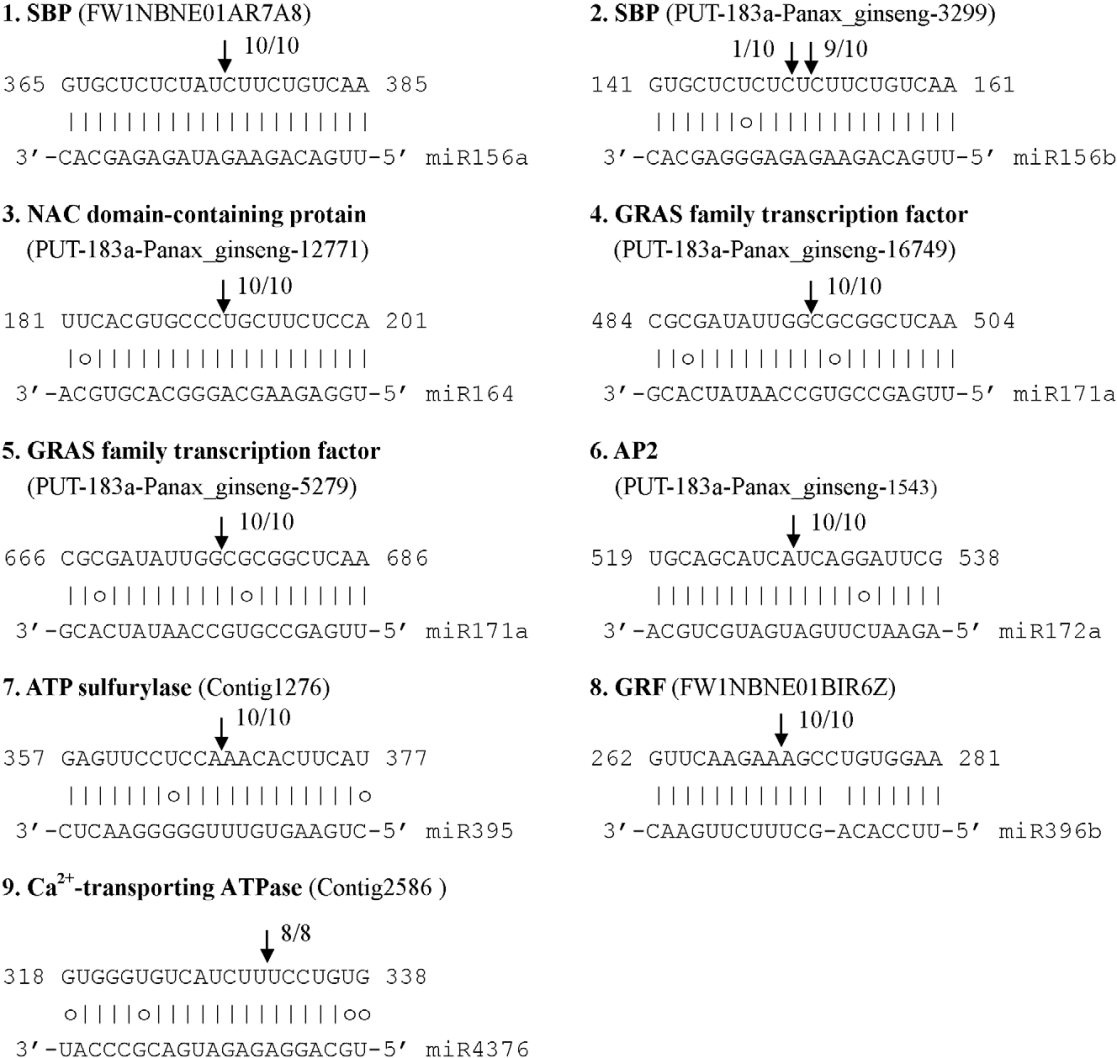
Validation of the predicted mRNA targets. The cleavage sites were determined by 5′ RLM-RACE. The nucleotide positions of the miRNA complementary sites of unigenes are indicated. The unigene sequence of each complementary site from 5′ to 3′ and the cloned miRNA sequence from 3′ to 5′ are shown. Watson-Crick pairing (vertical dashes) and G:U wobble pairing (circles) are indicated. Vertical arrows indicate the 5′ termini of miRNA-guided cleavage products, as identified by 5′ RLM-RACE, with the frequency of clones shown.

Twenty six of the 99 predicted targets are conserved among various plant species, suggesting the conserved regulatory roles of some miRNAs in plants (Table S4). Most of the conserved targets are transcription factor genes, including six squamosa promoter-binding-like protein genes (*SPLs*) regulated by miR156, four miR167-targeted auxin response factor genes (*ARFs*), three GRAS family transcription factor genes regulated by miR171, two homeobox-leucine zipper protein genes cleaved by miR166, and each of the NAC-domain protein gene, CCAAT-box binding factor gene, AP2 domain-containing protein gene and growth-regulating factor gene targeted by miR164, miR169, miR172, and miR396, respectively. In addition to transcription factor genes, the other seven conserved targets include four F-box family protein genes regulated by miR393 or miR394, an ATP sulfurylase gene cleaved by miR395, a laccase gene targeted by miR397, and a miR2118-regulated TIR-NBS-LRR resistance protein gene. Using the modified 5′ RLM-RACE method, we experimentally validated eight conserved targets of six miRNA gene families in *P. ginseng*, confirming the functional conservation of some miRNAs (Figure 4, Table S4).

Consistent with previous reports showing species-specific regulatory roles of many conserved miRNAs in various plant species, such as *Populus trichocarpa*, *Pinus taeda* and *Digitalis purpurea* [28], [35], [85], [97], seventy three of the 99 predicted miRNA targets are not conserved in other plants (Table S4). Among them, sixty could be important in plant development and defense responses, whereas the other thirteen are function-unknown. Using the modified 5′ RLM-RACE method, we experimentally validated contig2586, which encodes the Ca^2+^-transporting ATPase, to be a target of *MIR4376*. It suggests that conserved miRNAs may play species-specific functions by the cleavage of non-conserved targets.

### Targets of Non-conserved miRNAs

Using the computational approach, we predicted 31 targets for seven of nine non-conserved miRNA gene families (Table 2). Many of the predicted targets are associated with metabolism, signal transduction and stress responses, and about one-third of the targets encode proteins with unknown functions. These results are consistent with our previous findings from *P. trichocarpa* and confirm that a significant fraction of non-conserved miRNAs are part of the regulatory networks associated with specific growth conditions or developmental processes [35]. Interestingly, non-conserved miR6139 and miR1439 conserved between rice and *P. ginseng* were predicted to target the ZIP metal ion transporter gene (PUT-183a-Panax_ginseng-20083) for cleavage (Table 2 and Table S4), suggesting both miR6139 and miR1439 are probably involved in the homeostasis of metal ion and play a role in plant response to salt stress [98], [99]. Genes to be targeted by more than one miRNAs have been previously observed in other plant species [28]. For instance, five disease resistance protein genes were found to be targets of miR472, miR482 and/or miR1448, and fourteen pentatricopeptide repeat-containing protein (PPR) genes were regulated by both miR475 and miR476 in *P. trichocarpa* [28]. Because of the capability in cleaving the same target gene, miR6139 and miR1439 are probably members of a regulatory network.

**Table 2:** Targets of non-conserved miRNAs in *Panax ginseng*.

miRNA family	Target function	Unigene ID (penalty score)
MIR6135	Zinc finger CCCH-type antiviral protein	FW1NBNE01BOG42(1.5)
	β-Cyanoalanine synthase	PUT-183a-Panax_ginseng-3189(2.5)
	Mapkkk3	PUT-183a-Panax_ginseng-5059(3)
	Unknown	FW1NBNE01CDUDE(1.5), Contig2012(2.5), PUT-183a-Panax_ginseng-2440(3)
MIR6136	Unknown	PUT-183a-Panax_ginseng-1090(3), PUT-183a-Panax_ginseng-11673(3)
MIR6137	Armadillo/beta-catenin-like	PUT-183a-Panax_ginseng-3440(2.5)
	Protein phosphatase	PUT-183a-Panax_ginseng-4655(2.5)
	Repeat-containing protein	PUT-183a-Panax_ginseng-8005(3)
	Unknown	Contig872(3)
MIR6138	Asparagine synthetase	FW1NBNE01BSZXK(2)
MIR6139	Serine/threonine-protein phosphatase	FW1NBNE01BWTI7(2.5)
	ZIP transporter	PUT-183a-Panax_ginseng-20083(2.5)
	3-Hydroxybutyryl-CoA dehydratase	Contig1795(3)
	UDP-sugar transporter	PUT-183a-Panax_ginseng-9068(3)
	Unknown	FW1NBNE01B23TH(2.5), FW1NBNE01AQA9Q(2.5)
MIR6140	Phytanoyl-CoA dioxygenase-like protein	PUT-183a-Panax_ginseng-6406(2.5)
	KH domain-containing protein	PUT-183a-Panax_ginseng-11439(2.5)
	Casein kinase 2 subunit beta	PUT-183a-Panax_ginseng-10659(2.5)
	Villin	PUT-183a-Panax_ginseng-2502(3)
	Mitochondrial chaperonin-60	PUT-183a-Panax_ginseng-18641(3)
	DnaJ-like protein	FW1NBNE01CCFHV(3)
	Phytochrome	FW1NBNE01A44YJ(3)
	Unknown	PUT-183a-Panax_ginseng-19392(3), PUT-183a-Panax_ginseng-13359(3)
MIR6143	Homeobox-leucine zipper protein	Contig703(2.5)
	Acyl-CoA dehydrogenase	FW1NBNE01BR56P(3)
	Unknown	Contig1700(3)

### Tissue-specific Expression of miRNAs in *P. ginseng*

**Figure 5:**
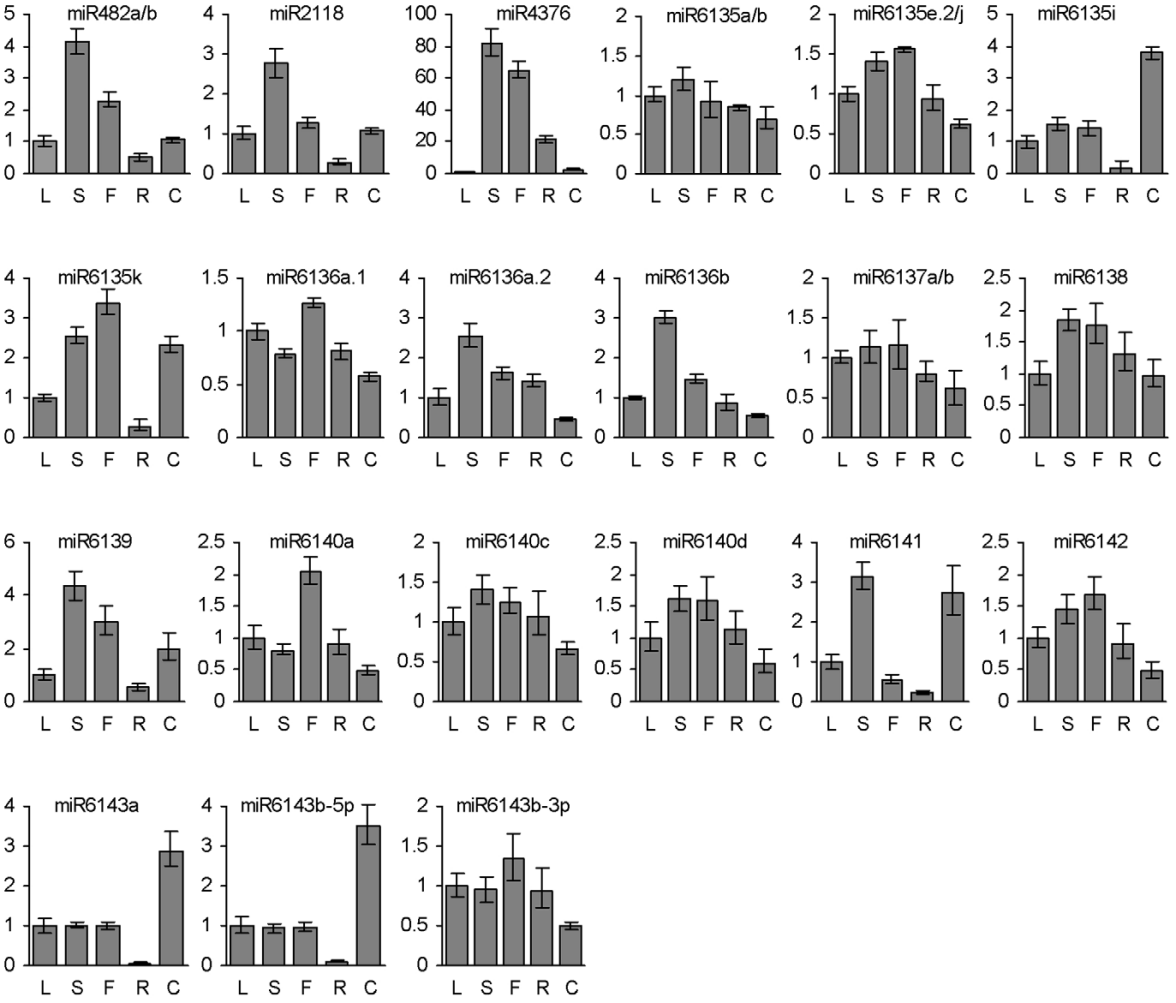
Tissue-specific expression of miRNAs in *P. ginseng*. Fold changes of miRNAs in leaves (L), stems (S), flowers (F), roots (R) of five-year-old *P. ginseng* plants and embryogenic calli (C) induced from the cotyledons of *P. ginseng* seeds are shown. miRNAs were analyzed using the poly(T) adaptor RT-PCR method. miRNA levels in leaves were arbitrarily set to 1.

In order to further predict the functions of miRNAs in *P. ginseng*, we analyzed the expression of mature miRNAs with known precursor sequences, including conserved miR482a/b, miR2118 and miR4376 and all of non-conserved miRNAs, in leaves, stems, flowers, roots, and embryogenic calli using the poly(A) miRNA-based qRT-PCR method as described [28], [35], [100], [101]. This method verifies the specificity of PCR products by single peak of dissociation curves and can readily discriminate the expression of mature miRNAs having as few as only one nucleotide difference [101]. As a result, the majority of analyzed mature miRNAs were specifically PCR-amplified and many of them showed tissue-specific expression patterns (Figure 5). For example, miR6135i, miR6143a and miR6143b-5p exhibited the highest expression in embryogenic calli, indicating their potential roles in embryogenic callus development and/or maintenance. On the contrary, miR4376, miR6135e,2/j, miR6136a.1, miR6136a.2, miR6136b, miR6140a, miR6140c, miR6140d and miR6142 showed low expression in embryogenic calli, whereas their expression was relatively high in stems and/or flowers. Differential expression was also found for many other miRNAs analyzed (Figure 5). It indicates tissue-specific regulation of miRNA expression in *P. ginseng*.

miR482a/b, miR2118 and miR6139 showed very similar expression patterns with the highest in stems, followed by flowers and roots (Figure 5). Among them, miR482a and miR2118 are generated from two precursors clustering in the same unigene PUT-183a-Panax_ginseng-17125 (Figure 3A). Similar expression patterns were previously observed for other clustered miRNAs that regulated same genes or different transcripts encoding functionally related proteins [77], [102], [103]. Consistently, both miR482 and miR2118 were predicted to target genes encoding disease resistance proteins (Table S4). It indicates that miR482a and miR2118 are involved in relevant cellular processes in *P. ginseng*.

miR6135i and miR6135e.2/j are members of a miRNA family. However, their expression patterns are distinct. miR6135i showed the highest expression in embryogenic calli, the lowest expression in roots, and moderate expression in stems and flowers, whereas miR6135e.2/j exhibited the highest expression in flowers, followed by stems, leaves and roots, and showed the lowest expression in embryogenic calli (Figure 5). It suggests that miR6135i and miR6135e.2/j could be functionally different in *P. ginseng*, although they belong to the same gene family. Interestingly, the expression patterns were also different between miR6136a.1 and miR6136a.2, two miRNAs generated from distinct regions of a precursor, FW1NBNE01BEGNB (Figure 5). Similarly, distinct expression patterns were observed for miR6143b-5p and miR6143b-3p produced from the unigene PUT-183a-Panax_ginseng-16660 (Figure 5). These results indicate that the level of miRNAs is not only associated with the transcription of primary miRNAs but also related to the maturation process and/or degradation rate of mature miRNAs. The expression pattern of miR6136a.2 is similar to miR6136b, a miRNA generated from the antisense transcript of FW1NBNE01BEGNB (Figure 5). It implies the existence of functional relationship between miR6136a.2 and miR6136b.

### Responses of Non-conserved miRNAs to Abiotic Stresses

**Figure 6:**
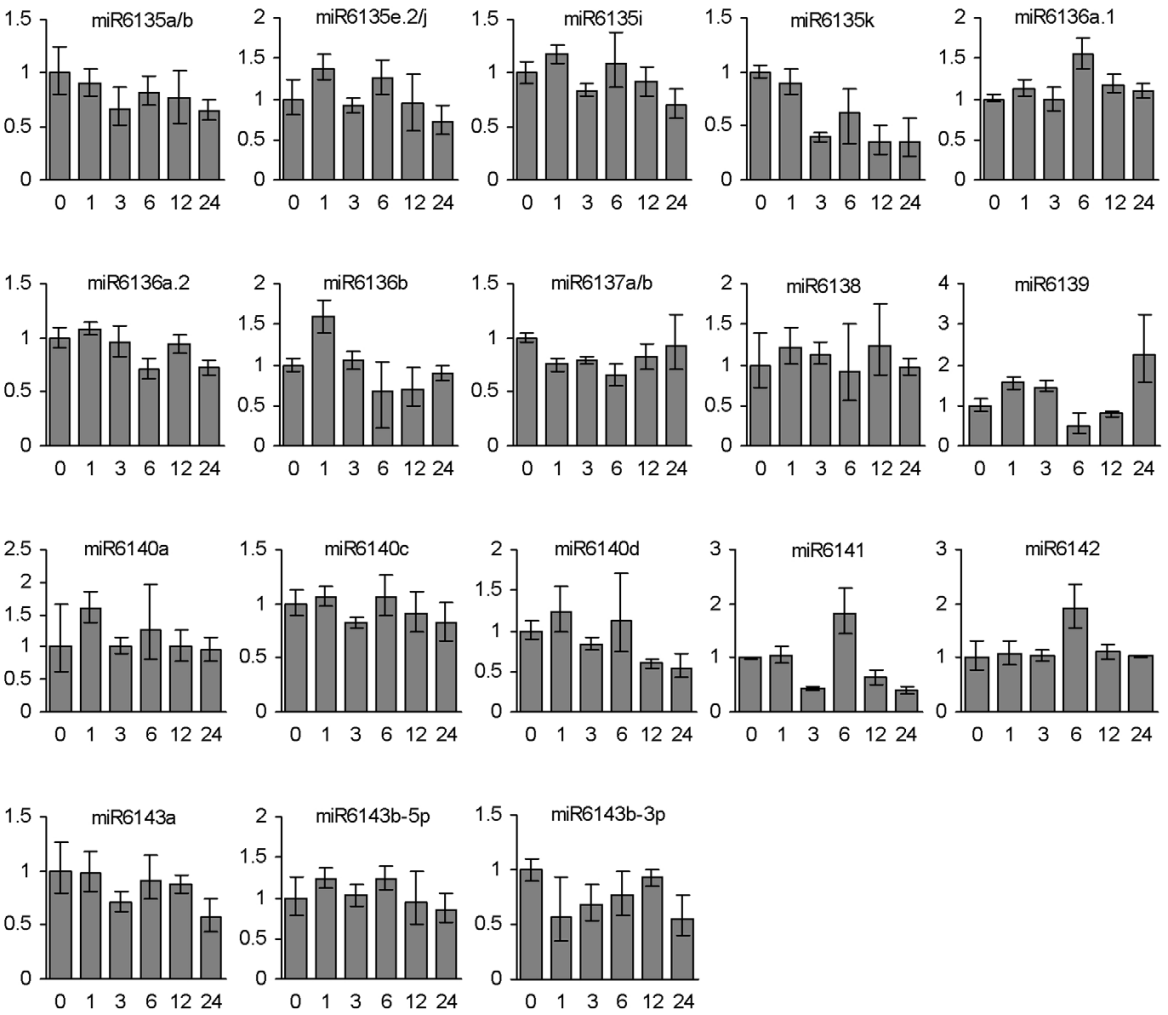
Responses of non-conserved miRNAs to dehydration stress. Fold changes of non-conserved miRNAs in ginseng embryogenic calli treated with dehydration for 0, 1, 3, 6, 12 and 24 h are shown. miRNAs were analyzed using the poly(T) adaptor RT-PCR method. miRNA levels in calli treated for 0 h were arbitrarily set to 1.

**Figure 7:**
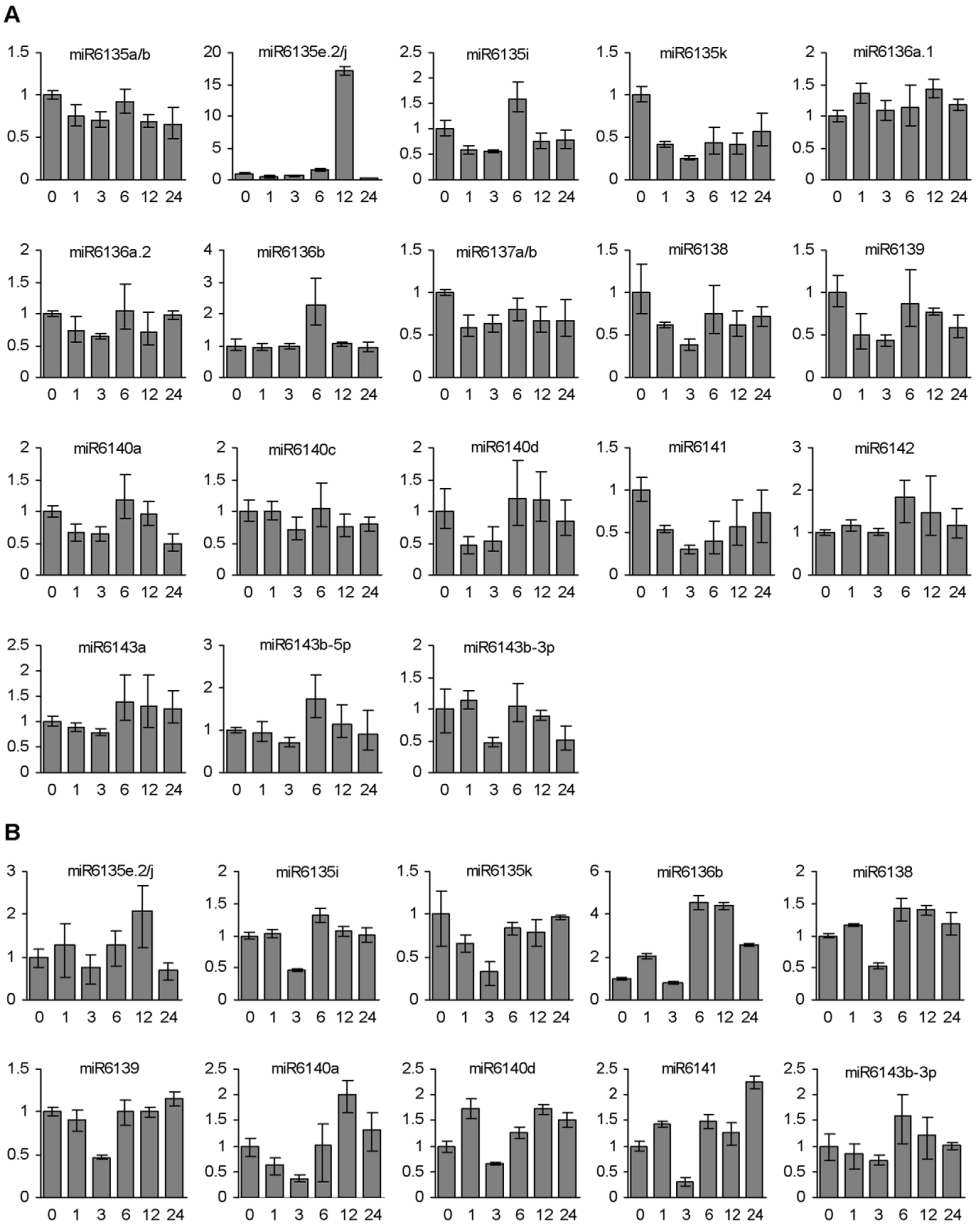
Responses of non-conserved miRNAs to heat stress. Fold changes of non-conserved miRNAs in ginseng embryogenic calli treated with heat (37°C) for 0, 1, 3, 6, 12 and 24 h are shown. miRNAs were analyzed using the poly(T) adaptor RT-PCR method (A) and the stem-loop RT-PCR method (B). miRNA levels in calli treated for 0 h were arbitrarily set to 1.

*In vitro* cultured embryogenic callus has been proved to be a great model for studying the role of miRNAs in meristem development, embryogenesis and abiotic stress responses in various plant species, such as rice [104], [105], Japanese Larch [106], and sweet orange [107]. To test whether non-conserved miRNAs are involved in plant response to environmental stresses, we analyzed the expression patterns of non-conserved miRNAs in embryogenic calli treated with dehydration and heat, two significant stressors experienced during the growth and development of *P. ginseng*, using the miRNA-specific ploy(T) adaptor RT-PCR method [101]. As a result, we identified five dehydration- and ten heat-responsive miRNAs, which showed more than two-fold changes between at least two time-points treated with dehydration and heat, respectively (Figures 6 and 7A). Among them, miR6135e.2/j, miR6135i, miR6138, miR6140a and miR6143b-3p responded to heat treatment only, whereas the other five, including miR6136b, miR6135k, miR6139, miR6140d, and miR6141, responded to dehydration and heat. It suggests the existence of a crosstalk among some non-conserved miRNAs in response to dehydration and heat stresses.

Due to low expression levels of non-conserved miRNAs, we used the other precise and sensitive miRNA-specific stem-loop RT-PCR method to further verify the expression patterns of ten heat-responsive miRNAs [108], [109]. The results suggest that the expression patterns of miRNAs from stem-loop RT-PCR and poly(T) adaptor RT-PCR are largely consistent (Figure 7B), validating the ploy(T) adaptor RT-PCR method in analysis of miRNA expression. Moreover, we cloned and sequenced some PCR amplicons. All of the miRNAs analyzed were found to be specifically amplified and had integral 3′ ends (Table S5). These results verify the specificity of PCR-amplification and suggest the integrity of miRNAs in the samples analyzed.

Among the five dehydration-responsive miRNAs, miR6315k was down-regulated after stress. The expression of miR6136b, miR6139, miR6140d and miR6141 was fluctuated (Figure 6). Similarly, different expression patterns were observed for ten heat-responsive miRNAs. miR6135i and miR6136b were induced after heat treatment for 6 h. miR6135e.2/j was highly up-regulated at 12 h after stress. However, the expression of miR6135k, miR6138, miR6139 and miR6141 was down-regulated after treatment. The expression of miR6140a, miR6140d and miR6143-3p was fluctuated in response to heat stress (Figure 7). These results imply that the underlying biological functions vary among stress-responsive miRNAs.

### Analysis of Other Small RNAs

In order to characterize other small RNAs, we mapped the remaining 1,502,563 unique small RNA sequences to 31,371 *P. ginseng* EST sequences using the SOAP2 program with no mismatches allowed and then searched for EST sequences with small RNA mapped [40]. Among the 31,371 ESTs, 14,743 have corresponding small RNAs. 77.65% of 14,743 generated less than five small RNAs. About 2% (294 ESTs) produced more than 50 unique small RNAs. It includes 18 with more than 400 unique small RNAs mapped. Among them, eleven produced from rRNAs, three from chloroplast genome, and one from mitochondrion DNA. The other three were predicted to encode ginsenoside biosynthesis-related proteins [110]. It suggests the complexity of small RNAs in *P. ginseng*. Full-length cDNA cloning and transgenic analysis may help to further characterize the small RNAs.

**Figure 8:**
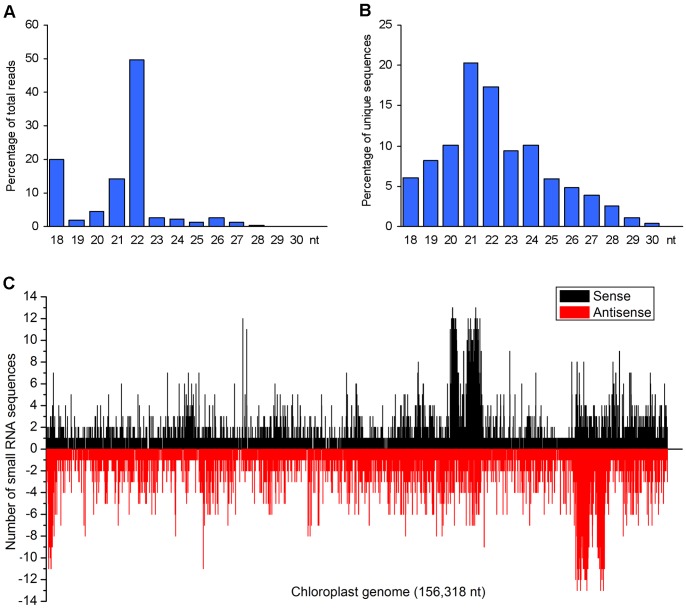
Analysis of chloroplast-derived small RNAs. (A) Size distribution of clean reads. (B) Size distribution of unique sequences. (C) The number of small RNA sequences mapped onto *P. ginseng* chloroplast genome. X-axis shows nucleotide positions of *P. ginseng* chloroplast genome. Y-axis shows the number of sRNAs mapped at each position. Black represents sense small RNAs, Red represents antisense small RNAs. The number of antisense sRNAs is shown as negative numbers.

The availability of whole genome sequence of *P. ginseng* chloroplast enabled us to further characterize the small RNAs derived from chloroplast [16]. Blast analysis of 1,502,563 unique small RNA sequences against the chloroplast genome showed that 45,971 small RNAs, representing 2,520,635 clean reads, generated hits over the chloroplast genome. Size distribution of clean reads revealed the 22 nt group, which represents 49.69% of the 2,520,635 reads, to be the biggest size group of chloroplast-derived small RNAs (Figure 8A). The reads of the 24 nt group account for 2.11% of the 2,520,635 reads, which is significantly less compared with the percentage of the 24 nt group in the total 12,000,591 clean reads (Figure 1A). Size distribution analysis of the 45,971 unique small RNA sequences showed the 21 nt group and the 22 nt group, which account for 20.33% and 17.32% of the total unique small RNA sequences, respectively, to be two major groups (Figure 8B). Further examination of the chloroplast-derived small RNAs revealed that they were mapped to both sense and antisense orientation in the *P. ginseng* chloroplast genome (Figure 8C). There are two clusters of small RNAs abundant in the loci annotated as chloroplast rRNAs, which are located in the inverted repeats (IRa and IRb) of chloroplast genome [16]. These results suggest the diversity of chloroplast-derived small RNAs in *P. ginseng*. Chloroplast-derived small RNAs have been previously identified in various species, such as tobacco [111] and *Cucumi*s [112]. In consistence with our results, two clusters of small RNAs derived from chloroplast rRNAs were also observed in melon, suggesting chloroplast small RNAs may be highly conserved.

## Discussion

The estimated genome of *P. ginseng* is about 3.12×10^3^ million letters of genetic code. It is 21.5 and 7.43 times of the genome of *Arabidopsis* and rice, respectively, indicating that the *P. ginseng* genome could be much more complicated than many other plant genomes [113], [114]. Consistently, we found in this study that a large, diverse and complex small RNA population existed in *P. ginseng*. Moreover, the population of *P. ginseng* small RNAs identified is far from exhausted because 88.5% of the total 1,502,664 unique small RNA sequences were sequenced only once or twice. Analysis of the size distribution of *P. ginseng* small RNAs revealed that the 24 nt group, which mainly contains repeat-associated siRNAs (ra-siRNAs) and transposable element-derived siRNAs (TE-siRNAs), is the most diverse (Figure 1), indicating the existence of a large number of repetitive DNA and transposons in the *P. ginseng* genome [115]–[117]. Many repetitive elements are located in the heterochromatic regions including centromeres and telomeres. ra-siRNAs generated from heterochromatic regions play significant roles in developing or/and maintaining the repressed chromatin state through DNA methylation and heterochromatic histone modification [55], [118]–[120]. It suggests that the existence of a complex and diverse small RNA population is particularly important to maintain the growth and development of plants, such as *P. ginseng*, which have a large genome.

From the *P. ginseng* small RNA data set, we identified a total of 73 conserved and 28 non-conserved miRNAs, which belong to 33 and 9 miRNA families, respectively. However, only four precursors of conserved miRNAs were identified from total 31,371 unigenes, suggesting the EST sequences available are far from sufficient for the identification of miRNA precursors. On the other hand, we were able to identify 25 non-conserved miRNA precursors from the same unigene set. It indicates that the number of non-conserved miRNAs is probably much larger than that of conserved miRNAs in *P. ginseng*. Among four precursors for conserved miRNAs, two (MIR482a and MIR2118) cluster in a transcript (Figure 3). Both miR482 and miR2118 were predicted to regulate disease resistance proteins (Table S4). Consistently, the expression patterns of miR482 and miR2118 are similar in the tissues analyzed. It suggests that miR482 and miR2118 are involved in defense responses in *P. ginseng* through relevant regulatory networks.

Among the non-conserved miRNAs, miR6136a.1 and miR6136a.2 are generated from the same precursor FW1NBNE01BEGNB, whereas its antisense transcript produces miR6136b. miR6136a.1 showed the highest expression in flowers, followed by leaves, whereas miR6136a.2 and miR6136b were mainly expressed in stems (Figure 5). The expression of miR6136b was altered in embryogenic calli treated with dehydration and heat, whereas both miR6136a.1 and miR6136a.2 appeared to be not responsive to the stresses (Figures 6 and 7). These results suggest the complexity of miRNA regulatory networks in *P. ginseng*.

Environmental factors are critical to plant growth and development. Increasing evidence indicates that miRNAs are involved in plant response to various environmental stresses, such as drought, salinity, cold, nutrient starvation, oxidative stress, mechanical stress and UV-B radiation [28], [30], [35], [100], [121]–[132]. In this work, we examined the expression profiles of non-conserved miRNAs under dehydration and heat treatments and identified 5 dehydration- and 10 heat-responsive *P. ginseng* miRNAs, which appeared to be an incomplete set of dehydration and heat-responsive miRNAs in *P. ginseng*, considering that the small RNAs analyzed in this study were isolated from plants growing under normal conditions. More stress-responsive miRNAs could be identified if plant tissues treated with dehydration and heat were used. Additionally, we found that a crosstalk could exist among some miRNAs in ginseng response to dehydration and heat stresses. However, the underlying mechanism remains to be elucidated.

## Materials and Methods

### Plant Materials

*P. ginseng* C.A. Meyer cv. Shizhu was grown in a field nursery at Kuandian, Liaoning province, China. Leaves, stems, flowers and roots were collected from five-year-old plants and frozen in liquid nitrogen for total RNA extraction and small RNA library construction. No specific permits were required for the described field studies. The location of the field nursery is not privately-owned or protected in any way and the field studies did not involve endangered or protected species. Embryogenic callus was induced as described [133]. Briefly, ginseng seeds were harvested and then stratified for about 6 months. Pericarp was removed before sterilization in 70% ethanol for 1 min and 1% sodium hypochlorite for 20 min. After washing with sterile water, cotyledon explants were dissected out from seeds and cultured on MS media containing 1.0 mg.L^-1^ 2,4-D and 3% sucrose in the dark for one month. Explants with embryogenic calli were then cultured at 24±2°C under a 16/8-h (light/dark) photoperiod with light supplied by white fluorescent tubes at an intensity of 24 µmol.m^-2^.s^-1^. Embryogenic calli were maintained by regular 3-week subcultures.

### Stress Treatments

For dehydration treatment, embryogenic calli were transferred to a 9 cm Petri dish with a dry sterile filter paper. The Petri dish was sealed with Parafilm and kept in a clean bench for 0, 1, 3, 6, 12 and 24 h at 24°C. For heat treatment, the calli on a callus induction medium were incubated at 37°C in the dark for 0, 1, 3, 6, 12 and 24 h. After each treatment, the calli were collected and blotted briefly with a piece of dry filter paper. Tissues were immediately stored in liquid nitrogen until use.

### Small RNA Library Construction and High-throughput Sequencing

Total RNA was extracted from leaves, stems, flowers and roots of *P. ginseng* using the TRIzol Reagent (Invitrogen, USA). Equal amounts (5 µg) of total RNA from each tissue were pooled. The quality and quantity of total RNA were analyzed using Agilent 2100 (Figure S2). Small RNA library was prepared as described [134]. Briefly, 10–30 nt small RNAs were purified from a 15% denaturing polyacrylamide gel and then ligated with the 5′ and 3′ adapters. After being reverse-transcribed by Superscript II reverse transcriptase (Invitrogen, USA), small RNAs were amplified by PCR. High-throughput sequencing of small RNAs was performed using an Illumina G1 Genome Analyzer at BGI-Shenzhen, China.

### Electrophoretic Analysis of Small RNAs

Low molecular weight (LMW) RNA from 200 µg pooled total RNA was resolved on a 15% denaturing polyacrylamide gel and stained with ethidium bromide as described by Lu et al. [49]. The pictures were taken with the Bio-Rad Universal Hood II Gel Imager.

### Data Sets

The 20621 PlantGDB-assembled unique transcripts (PUTs) of *P. ginseng* were downloaded from PlantGDB (http://www.plantgdb.org, April 25th, 2011) and further assembled with singletons identified previously by EGassembler using the default parameters (http://egassembler.hgc.jp/) [13], [135]. The resulting 31371 unigenes were then used for small RNA mapping. Known mature miRNAs and miRNA precursors were downloaded from miRBase for the analysis of conserved miRNAs in *P. ginseng* [38].

### Computational Identification of Small RNAs

Adapters were removed and low-quantity sequences were filtered from raw small RNA sequence data using the PHRED and CROSS MATCH programs [136]. The 18–30 nt clean small RNAs were mapped to known miRNA precursors using the SOAP2 program with no more than 2 mismatches allowed [40]. Small RNAs with matched precursors were then checked for mature miRNAs and miRNA* manually. For identification of non-conserved miRNAs, two approaches were used. First, small RNAs were mapped to 31371 unigenes of *P. ginseng* using the SOAP2 program with no mismatches allowed [40]. Unigenes with small RNA mapped were analyzed for secondary structures using the mfold program [75]. Second, small RNAs were mapped to *P. ginseng* unigenes and miRNAs were predicted using the MIREAP software (http://sourceforge.net/projects/mireap/) with the following parameters applied. It includes minimal miRNA length (18), maximal miRNA length (26), minimal miRNA (reference) length (20), maximal miRNA reference length (24), uniquencess of miRNA (280), maximal energy (-30), minimal space (5), maximal space (450), minimal mature pair (16), maximal mature bulge (3), maximal duplex asymmetry (4), and flank sequence length (20). The resulting hairpin structures from both approaches were then manually checked. The criteria applied to manual miRNA annotation includes (1) at least 2 reads for miRNAs, (2) a single-stranded, hairpin precursor with the free energy (dG) value less than -30, (3) four or fewer mismatched miRNA bases suggested by Meyers et al (2008) [137], (4) no more than two asymmetric bulges within the miRNA/miRNA* duplex, and (5) at least 0.85 of the minimal folding free energy index (MFEI) calculated as described [138].

### Computational Prediction, Annotation and Experimental Validation of miRNA Targets

miRNA targets were predicted from the assembled *P. ginseng* unigene set by the psRNATarget server using default parameters [95]. Unigenes were annotated by blastx analysis against the Nr protein database using default parameters (http://www.ncbi.nlm.nih.gov/). The transcriptional direction of unigenes was determined based on the direction of predicted proteins. Unigenes without a hit in the Nr protein database were not considered as miRNA targets because the transcriptional direction was unknown without further experiments. Experimental validation of miRNA targets were carried out on mRNAs isolated from leaves or pooled tissues of leaves, stems, flowers and roots of *P. ginseng* using the modified 5′ RNA ligase-mediated (RLM)-RACE method as described previously [35]. The nesting and nested primers used for PCR were listed in Table S6.

### Quantitative Real-time Reverse Transcription-PCR (qRT-PCR) Analysis of miRNAs

Total RNA was isolated from leaves, stems, flowers, roots and calli using the TRIzol Reagent (Invitrogen, USA) and digested with RNase-free DNase I (Promega, USA) to remove genomic DNA. Two qRT-PCR methods, including the poly(T) adaptor RT-PCR method [101] and the stem-loop RT-PCR method [108], [109], were used for miRNA quantification. The poly(T) adaptor RT-PCR method used about 1 µg DNase-treated total RNA as the starting material, which was polyadenylated using the Poly(A) Tailing kit (Ambion, USA) as described previously [101] and then reverse-transcribed into single-strand cDNA in a 20 µl reaction mix with SuperScript III Reverse Transcriptase (Invitrogen, USA) and 0.5 µg Poly(T) adapter (5′-GCGAGCACAGAATTAATACGACTCACTATAGG(T)_12_VN-3′, Ambion, USA). qRT-PCRs were performed in triplicates using the Bio-Rad CFX96 Real-Time PCR System C1000 Thermal Cycler (Bio-Rad, USA). Each PCR reaction was carried out in a final volume of 20 µl containing 10 µl 2×SYBR Premix Ex Taq II (TaKaRa, Japan), 0.25 µM each of the miRNA specific forward primer and the universal reverse primer (Table S7), and the cDNA reverse-transcribed from 100 pg total RNA using the following conditions: 95°C for 20 sec, 40 cycles of 95°C for 15 sec, 60°C for 15 sec and 72°C for 15 sec. 5.8 S rRNA was used as an endogenous reference as described previously [28], [35], [100], [101]. Standard deviations were calculated from three PCR replicates. The specificity of PCR products was verified based on single peak of dissociation curves. The relative abundance of miRNAs was determined using the 2^-ΔΔCq^ method, where Cq represents the threshold cycle [139].

For the stem-loop RT-PCR method, about 100 ng DNA-free total RNA was hybridized with a miRNA-specific stem-loop RT primer. The hybridized miRNA molecules were then reverse-transcribed into cDNA as described [108], [109]. cDNA was diluted and then PCR-amplified in triplicates using the Bio-Rad CFX96 Real-Time PCR System C1000 Thermal Cycler (Bio-Rad, USA). Each PCR reaction was performed in a final volume of 20 µl containing 10 µl 2×SYBR Premix Ex Taq II (TaKaRa, Japan), 0.25 µM each of the miRNA specific forward primer and the universal reverse primer (Table S8), and the cDNA reverse-transcribed from 10 ng total RNA using the following conditions: 95°C for 20 sec, 40 cycles of 95°C for 10 sec, 60°C for 10 sec and 72°C for 6 sec. The endogenous reference, 5.8 S rRNA, was analyzed on the cDNA template converted from total RNA using random primers (Invitrogen, USA). In order to verify the specificity of PCR-amplification, some amplicons were cloned and sequenced.

## Supporting Information

Figure S1 **Predicted hairpin structures of** ***P. ginseng*** **miRNA precursors.** Mature miRNA sequences are indicated in red. miRNA* sequences are indicated in blue.
(DOC)

Figure S2 **The quality and quantity of total RNA used for small RNA library construction.** One µl of total RNA were diluted with 1 µl of RNase-free water and then denatured at 70°C for two min. Total RNA was analyzed using Agilent 2100. The results showed that the sample had a RNA integrity number (RIN) of 8.1 and a 28s/18s rRNA ratio of 1.8, suggesting the RNA samples were not degraded. The concentration of original total RNA is 302 ng/µl, which is good for small RNA library construction.
(DOC)

Table S1 **Statistics of high-throughput sequencing results of** ***P. ginseng*** **small RNAs.**
(DOC)

Table S2 **Conserved miRNAs in** ***P. ginseng*** **.**
(DOC)

Table S3 miRNAs* putatively produced from conserved miRNA gene families in ***P. ginseng*** **.**
(DOC)

Table S4 **Targets of conserved miRNAs in** ***P. ginseng*** **.**
(DOC)

Table S5 **Sequencing analysis of miRNA amplicons from stem-loop RT-PCR.**
(DOC)

Table S6 **Primers used for 5′ RLM-RACE.**
(DOC)

Table S7 **Primers used for poly(T) adaptor RT-PCR.**
(DOC)

Table S8 **Primers used for stem-loop RT-PCR.**
(DOC)

## References

[1] Chong SKF, Oberholzer VG (1988) Gingseng-is there a use in clinical medicine? Postgraduate Medical Journal 64: 841–846. 3076665 10.1136/pgmj.64.757.841 PMC2429058

[2] Dey L, Xie JT, Wang A, Wu J, Maleckar SA, et al (2003) Anti-hyperglycemic effects of ginseng: comparison between root and berry. Phytomedicine 10: 600–605. 13678250 10.1078/094471103322331908

[3] Larsen MW, Moser C, Hoiby N, Song Z, Kharazmi A (2004) Ginseng modulates the immune response by induction of interleukin-12 production. APMIS 112: 369–373. 15511274 10.1111/j.1600-0463.2004.apm1120607.x

[4] Lee MH, Jeong JH, Seo JW, Shin CG, Kim YS, et al (2004) Enhanced triterpene and phytosterol biosynthesis in *Panax ginseng* overexpressing squalene synthase gene. Plant Cell Physiol 45: 976–984. 15356323 10.1093/pcp/pch126

[5] O’Hara M, Kiefer D, Farrell K, Kemper K (1998) A review of 12 commonly used medicinal herbs. Arch Fam Med 7: 523–536. 9821826 10.1001/archfami.7.6.523

[6] Shibata S (2001) Chemistry and cancer preventing activities of ginseng saponins and some related triterpenoid compounds. J Korean Med Sci 16 Suppl: S28–37 11748374 10.3346/jkms.2001.16.S.S28 PMC3202208

[7] Briskin DP (2000) Medicinal plants and phytomedicines. Linking plant biochemistry and physiology to human health. Plant Physiol 124: 507–514. 11027701 10.1104/pp.124.2.507 PMC1539282

[8] Dewick PM (2001) Medicinal Natural Products. West Sussex: JohnWiley & Sons Ltd press, 219p.

[9] World Health Organization (1999) WHO monographs on selected medicinal plants, Vol 1 Geneva: World Health Organization press, 171p.

[10] Ali MB, Hahn EJ, Paek KY (2005) CO(2)-induced total phenolics in suspension cultures of *Panax ginseng* C. A. Mayer roots: role of antioxidants and enzymes. Plant Physiol Biochem 43: 449–457. 15878284 10.1016/j.plaphy.2005.03.005

[11] Gao QP, Kiyohara H, Cyong JC, Yamada H (1989) Chemical properties and anti-complementary activities of polysaccharide fractions from roots and leaves of *Panax ginseng* . Planta Med 55: 9–12. 17262250 10.1055/s-2006-961765

[12] Shin HJ, Kim YS, Kwak YS, Song YB, Park JD (2004) Enhancement of antitumor effects of paclitaxel (taxol) in combination with red ginseng acidic polysaccharide (RGAP). Planta Med 70: 1033–1038. 15549658 10.1055/s-2004-832643

[13] Chen S, Luo H, Li Y, Sun Y, Wu Q, et al (2011) 454 EST analysis detects genes putatively involved in ginsenoside biosynthesis in *Panax ginseng* . Plant Cell Rep 30: 1593–1601. 21484331 10.1007/s00299-011-1070-6

[14] Choi DW, Jung J, Ha YI, Park HW, In DS, et al (2005) Analysis of transcripts in methyl jasmonate-treated ginseng hairy roots to identify genes involved in the biosynthesis of ginsenosides and other secondary metabolites. Plant Cell Rep 23: 557–566. 15538577 10.1007/s00299-004-0845-4

[15] Jung JD, Park HW, Hahn Y, Hur CG, In DS, et al (2003) Discovery of genes for ginsenoside biosynthesis by analysis of ginseng expressed sequence tag. Plant Cell Reports 22: 224–230. 12920566 10.1007/s00299-003-0678-6

[16] Kim KJ, Lee HL (2004) Complete chloroplast genome sequences from Korean ginseng (*Panax schinseng* Nees) and comparative analysis of sequence evolution among 17 vascular plants. DNA Res 11: 247–261. 15500250 10.1093/dnares/11.4.247

[17] Kim MK, Lee BS, In JG, Sun H, Yoon JH, et al (2006) Comparative analysis of expressed sequence tags (ESTs) of ginseng leaf. Plant Cell Rep 25: 599–606. 16397780 10.1007/s00299-005-0095-0

[18] Han JY, In JG, Kwon YS, Choi YE (2010) Regulation of ginsenoside and phytosterol biosynthesis by RNA interferences of squalene epoxidase gene in *Panax ginseng* . Phytochemistry 71: 36–46. 19857882 10.1016/j.phytochem.2009.09.031

[19] Han JY, Kwon YS, Yang DC, Jung YR, Choi YE (2006) Expression and RNA interference-induced silencing of the dammarenediol synthase gene in Panax ginseng. Plant Cell Physiol 47: 1653–1662. 17088293 10.1093/pcp/pcl032

[20] Bartel DP (2004) MicroRNAs: genomics, biogenesis, mechanism, and function. Cell 116: 281–297. 14744438 10.1016/s0092-8674(04)00045-5

[21] Jones-Rhoades MW, Bartel DP, Bartel B (2006) MicroRNAS and their regulatory roles in plants. Annu Rev Plant Biol 57: 19–53. 16669754 10.1146/annurev.arplant.57.032905.105218

[22] Mallory AC, Vaucheret H (2006) Functions of microRNAs and related small RNAs in plants. Nat Genet 38 Suppl: S31–36 16736022 10.1038/ng1791

[23] Brodersen P, Sakvarelidze-Achard L, Bruun-Rasmussen M, Dunoyer P, Yamamoto YY, et al (2008) Widespread translational inhibition by plant miRNAs and siRNAs. Science 320: 1185–1190. 18483398 10.1126/science.1159151

[24] Wu L, Zhou H, Zhang Q, Zhang J, Ni F, et al (2010) DNA methylation mediated by a microRNA pathway. Mol Cell 38: 465–475. 20381393 10.1016/j.molcel.2010.03.008

[25] Carrington JC, Ambros V (2003) Role of microRNAs in plant and animal development. Science 301: 336–338. 12869753 10.1126/science.1085242

[26] Hake S (2003) MicroRNAs: a role in plant development. Curr Biol 13: R851–852. 14588265 10.1016/j.cub.2003.10.021

[27] Kidner CA, Martienssen RA (2005) The developmental role of microRNA in plants. Curr Opin Plant Biol 8: 38–44. 15653398 10.1016/j.pbi.2004.11.008

[28] Lu S, Sun YH, Chiang VL (2008) Stress-responsive microRNAs in Populus. Plant J 55: 131–151. 18363789 10.1111/j.1365-313X.2008.03497.x

[29] Navarro L, Dunoyer P, Jay F, Arnold B, Dharmasiri N, et al (2006) A plant miRNA contributes to antibacterial resistance by repressing auxin signaling. Science 312: 436–439. 16627744 10.1126/science.1126088

[30] Sunkar R, Zhu JK (2004) Novel and stress-regulated microRNAs and other small RNAs from *Arabidopsis* . Plant Cell 16: 2001–2019. 15258262 10.1105/tpc.104.022830 PMC519194

[31] Aukerman MJ, Sakai H (2003) Regulation of flowering time and floral organ identity by a microRNA and its *APETALA2*-like target genes. Plant Cell 15: 2730–2741. 14555699 10.1105/tpc.016238 PMC280575

[32] Chen X (2004) A microRNA as a translational repressor of *APETALA2* in *Arabidopsis* flower development. Science 303: 2022–2025. 12893888 10.1126/science.1088060 PMC5127708

[33] Schmid M, Uhlenhaut NH, Godard F, Demar M, Bressan R, et al (2003) Dissection of floral induction pathways using global expression analysis. Development 130: 6001–6012. 14573523 10.1242/dev.00842

[34] Gutierrez L, Bussell JD, Pacurar DI, Schwambach J, Pacurar M, et al (2009) Phenotypic plasticity of adventitious rooting in *Arabidopsis* is controlled by complex regulation of *AUXIN RESPONSE FACTOR* transcripts and microRNA abundance. Plant Cell 21: 3119–3132. 19820192 10.1105/tpc.108.064758 PMC2782293

[35] Lu S, Sun YH, Shi R, Clark C, Li L, et al (2005) Novel and mechanical stress-responsive microRNAs in *Populus trichocarpa* that are absent from *Arabidopsis* . Plant Cell 17: 2186–2203. 15994906 10.1105/tpc.105.033456 PMC1182482

[36] Llave C, Kasschau KD, Rector MA, Carrington JC (2002) Endogenous and silencing-associated small RNAs in plants. Plant Cell 14: 1605–1619. 12119378 10.1105/tpc.003210 PMC150710

[37] Reinhart BJ, Weinstein EG, Rhoades MW, Bartel B, Bartel DP (2002) MicroRNAs in plants. Genes Dev 16: 1616–1626. 12101121 10.1101/gad.1004402 PMC186362

[38] Kozomara A, Griffiths-Jones S (2011) miRBase: integrating microRNA annotation and deep-sequencing data. Nucleic Acids Res 39: D152–157. 21037258 10.1093/nar/gkq1027 PMC3013655

[39] Quail MA, Kozarewa I, Smith F, Scally A, Stephens PJ, et al (2008) A large genome center’s improvements to the Illumina sequencing system. Nat Methods 5: 1005–1010. 19034268 10.1038/nmeth.1270 PMC2610436

[40] Li R, Yu C, Li Y, Lam TW, Yiu SM, et al (2009) SOAP2: an improved ultrafast tool for short read alignment. Bioinformatics 25: 1966–1967. 19497933 10.1093/bioinformatics/btp336

[41] Lu C, Tej SS, Luo S, Haudenschild CD, Meyers BC, et al (2005) Elucidation of the small RNA component of the transcriptome. Science 309: 1567–1569. 16141074 10.1126/science.1114112

[42] Szittya G, Moxon S, Santos DM, Jing R, Fevereiro MP, et al (2008) High-throughput sequencing of *Medicago truncatula* short RNAs identifies eight new miRNA families. BMC Genomics 9: 593. 19068109 10.1186/1471-2164-9-593 PMC2621214

[43] Zhang J, Xu Y, Huan Q, Chong K (2009) Deep sequencing of *Brachypodium* small RNAs at the global genome level identifies microRNAs involved in cold stress response. BMC Genomics 10: 449. 19772667 10.1186/1471-2164-10-449 PMC2759970

[44] Zhao CZ, Xia H, Frazier TP, Yao YY, Bi YP, et al (2010) Deep sequencing identifies novel and conserved microRNAs in peanuts (*Arachis hypogaea* L.). BMC Plant Biol 10: 3. 20047695 10.1186/1471-2229-10-3 PMC2826338

[45] Song C, Wang C, Zhang C, Korir NK, Yu H, et al (2010) Deep sequencing discovery of novel and conserved microRNAs in trifoliate orange (*Citrus trifoliata*). BMC Genomics 11: 431. 20626894 10.1186/1471-2164-11-431 PMC2996959

[46] Kwak PB, Wang QQ, Chen XS, Qiu CX, Yang ZM (2009) Enrichment of a set of microRNAs during the cotton fiber development. BMC Genomics 10: 457. 19788742 10.1186/1471-2164-10-457 PMC2760587

[47] Morin RD, Aksay G, Dolgosheina E, Ebhardt HA, Magrini V, et al (2008) Comparative analysis of the small RNA transcriptomes of *Pinus contorta* and *Oryza sativa* . Genome Res 18: 571–584. 18323537 10.1101/gr.6897308 PMC2279245

[48] Wang L, Liu H, Li D, Chen H (2011) Identification and characterization of maize microRNAs involved in the very early stage of seed germination. BMC Genomics 12: 154. 21414237 10.1186/1471-2164-12-154 PMC3066126

[49] Lu C, Meyers BC, Green PJ (2007) Construction of small RNA cDNA libraries for deep sequencing. Methods 43: 110–117. 17889797 10.1016/j.ymeth.2007.05.002

[50] Pantaleo V, Szittya G, Moxon S, Miozzi L, Moulton V, et al (2010) Identification of grapevine microRNAs and their targets using high-throughput sequencing and degradome analysis. Plant J 62: 960–976. 20230504 10.1111/j.0960-7412.2010.04208.x

[51] Herr AJ (2005) Pathways through the small RNA world of plants. FEBS Lett 579: 5879–5888. 16162339 10.1016/j.febslet.2005.08.040

[52] Mi S, Cai T, Hu Y, Chen Y, Hodges E, et al (2008) Sorting of small RNAs into *Arabidopsis* argonaute complexes is directed by the 5′ terminal nucleotide. Cell 133: 116–127. 18342361 10.1016/j.cell.2008.02.034 PMC2981139

[53] Onodera Y, Haag JR, Ream T, Costa Nunes P, Pontes O, et al (2005) Plant nuclear RNA polymerase IV mediates siRNA and DNA methylation-dependent heterochromatin formation. Cell 120: 613–622. 15766525 10.1016/j.cell.2005.02.007

[54] Rajagopalan R, Vaucheret H, Trejo J, Bartel DP (2006) A diverse and evolutionarily fluid set of microRNAs in *Arabidopsis thaliana* . Genes Dev 20: 3407–3425. 17182867 10.1101/gad.1476406 PMC1698448

[55] Xie Z, Johansen LK, Gustafson AM, Kasschau KD, Lellis AD, et al (2004) Genetic and functional diversification of small RNA pathways in plants. PLoS Biol 2: E104. 15024409 10.1371/journal.pbio.0020104 PMC350667

[56] Klevebring D, Street NR, Fahlgren N, Kasschau KD, Carrington JC, et al (2009) Genome-wide profiling of *Populus* small RNAs. BMC Genomics 10: 620. 20021695 10.1186/1471-2164-10-620 PMC2811130

[57] Mica E, Piccolo V, Delledonne M, Ferrarini A, Pezzotti M, et al (2009) High throughput approaches reveal splicing of primary microRNA transcripts and tissue specific expression of mature microRNAs in *Vitis vinifera* . BMC Genomics 10: 558. 19939267 10.1186/1471-2164-10-558 PMC2822795

[58] Zeng C, Wang W, Zheng Y, Chen X, Bo W, et al (2010) Conservation and divergence of microRNAs and their functions in Euphorbiaceous plants. Nucleic Acids Res 38: 981–995. 19942686 10.1093/nar/gkp1035 PMC2817462

[59] Zhang BH, Pan XP, Wang QL, Cobb GP, Anderson TA (2005) Identification and characterization of new plant microRNAs using EST analysis. Cell Res 15: 336–360. 15916721 10.1038/sj.cr.7290302

[60] Axtell MJ, Snyder JA, Bartel DP (2007) Common functions for diverse small RNAs of land plants. Plant Cell 19: 1750–1769. 17601824 10.1105/tpc.107.051706 PMC1955733

[61] Chen R, Hu Z, Zhang H (2009) Identification of microRNAs in wild soybean (*Glycine soja*). J Integr Plant Biol 51: 1071–1079. 20021554 10.1111/j.1744-7909.2009.00887.x

[62] Fattash I, Voss B, Reski R, Hess WR, Frank W (2007) Evidence for the rapid expansion of microRNA-mediated regulation in early land plant evolution. BMC Plant Biol 7: 13. 17359535 10.1186/1471-2229-7-13 PMC1838911

[63] Itaya A, Bundschuh R, Archual AJ, Joung JG, Fei Z, et al (2008) Small RNAs in tomato fruit and leaf development. Biochim Biophys Acta 1779: 99–107. 18078843 10.1016/j.bbagrm.2007.09.003

[64] Joshi T, Yan Z, Libault M, Jeong DH, Park S, et al (2010) Prediction of novel miRNAs and associated target genes in *Glycine max* . BMC Bioinformatics 11 Suppl 1: S14. 10.1186/1471-2105-11-S1-S14 PMC3009485 20122185

[65] Kulcheski FR, de Oliveira LF, Molina LG, Almerao MP, Rodrigues FA, et al (2011) Identification of novel soybean microRNAs involved in abiotic and biotic stresses. BMC Genomics 12: 307. 21663675 10.1186/1471-2164-12-307 PMC3141666

[66] Moxon S, Jing R, Szittya G, Schwach F, Rusholme Pilcher RL, et al (2008) Deep sequencing of tomato short RNAs identifies microRNAs targeting genes involved in fruit ripening. Genome Res 18: 1602–1609. 18653800 10.1101/gr.080127.108 PMC2556272

[67] Schreiber AW, Shi BJ, Huang CY, Langridge P, Baumann U (2011) Discovery of barley miRNAs through deep sequencing of short reads. BMC Genomics 12: 129. 21352554 10.1186/1471-2164-12-129 PMC3060140

[68] Subramanian S, Fu Y, Sunkar R, Barbazuk WB, Zhu JK, et al (2008) Novel and nodulation-regulated microRNAs in soybean roots. BMC Genomics 9: 160. 18402695 10.1186/1471-2164-9-160 PMC2335117

[69] Sunkar R, Zhou X, Zheng Y, Zhang W, Zhu JK (2008) Identification of novel and candidate miRNAs in rice by high throughput sequencing. BMC Plant Biol 8: 25. 18312648 10.1186/1471-2229-8-25 PMC2292181

[70] Yakovlev IA, Fossdal CG, Johnsen O (2010) MicroRNAs, the epigenetic memory and climatic adaptation in Norway spruce. New Phytol 187: 1154–1169. 20561211 10.1111/j.1469-8137.2010.03341.x

[71] Yang Y, Chen X, Chen J, Xu H, Li J, et al (2011) Differential miRNA expression in *Rehmannia glutinosa* plants subjected to continuous cropping. BMC Plant Biol 11: 53. 21439075 10.1186/1471-2229-11-53 PMC3078876

[72] Zhu QH, Spriggs A, Matthew L, Fan L, Kennedy G, et al (2008) A diverse set of microRNAs and microRNA-like small RNAs in developing rice grains. Genome Res 18: 1456–1465. 18687877 10.1101/gr.075572.107 PMC2527712

[73] Qiu D, Pan X, Wilson IW, Li F, Liu M, et al (2009) High throughput sequencing technology reveals that the taxoid elicitor methyl jasmonate regulates microRNA expression in Chinese yew (*Taxus chinensis*). Gene 436: 37–44. 19393185 10.1016/j.gene.2009.01.006

[74] ‘t Hoen PA, Ariyurek Y, Thygesen HH, Vreugdenhil E, Vossen RH, et al (2008) Deep sequencing-based expression analysis shows major advances in robustness, resolution and inter-lab portability over five microarray platforms. Nucleic Acids Res 36: e141. 18927111 10.1093/nar/gkn705 PMC2588528

[75] Zuker M (2003) Mfold web server for nucleic acid folding and hybridization prediction. Nucleic Acids Res 31: 3406–3415. 12824337 10.1093/nar/gkg595 PMC169194

[76] Fahlgren N, Howell MD, Kasschau KD, Chapman EJ, Sullivan CM, et al (2007) High-throughput sequencing of *Arabidopsis* microRNAs: evidence for frequent birth and death of MIRNA genes. PLoS One 2: e219. 17299599 10.1371/journal.pone.0000219 PMC1790633

[77] Altuvia Y, Landgraf P, Lithwick G, Elefant N, Pfeffer S, et al (2005) Clustering and conservation patterns of human microRNAs. Nucleic Acids Res 33: 2697–2706. 15891114 10.1093/nar/gki567 PMC1110742

[78] Tanzer A, Amemiya CT, Kim CB, Stadler PF (2005) Evolution of microRNAs located within *Hox* gene clusters. J Exp Zool B Mol Dev Evol 304: 75–85. 15643628 10.1002/jez.b.21021

[79] Tanzer A, Stadler PF (2004) Molecular evolution of a microRNA cluster. J Mol Biol 339: 327–335. 15136036 10.1016/j.jmb.2004.03.065

[80] Frazier TP, Xie F, Freistaedter A, Burklew CE, Zhang B (2010) Identification and characterization of microRNAs and their target genes in tobacco (*Nicotiana tabacum*). Planta 232: 1289–1308. 20803216 10.1007/s00425-010-1255-1

[81] Jin W, Li N, Zhang B, Wu F, Li W, et al (2008) Identification and verification of microRNA in wheat (*Triticum aestivum*). J Plant Res 121: 351–355. 18357413 10.1007/s10265-007-0139-3

[82] Zhang B, Pan X, Stellwag EJ (2008) Identification of soybean microRNAs and their targets. Planta 229: 161–182. 18815805 10.1007/s00425-008-0818-x

[83] Talmor-Neiman M, Stav R, Frank W, Voss B, Arazi T (2006) Novel micro-RNAs and intermediates of micro-RNA biogenesis from moss. Plant J 47: 25–37. 16824179 10.1111/j.1365-313X.2006.02768.x

[84] Xie F, Frazier TP, Zhang B (2010) Identification and characterization of microRNAs and their targets in the bioenergy plant switchgrass (*Panicum virgatum*). Planta 232: 417–434. 20461402 10.1007/s00425-010-1182-1

[85] Lu S, Sun YH, Amerson H, Chiang VL (2007) MicroRNAs in loblolly pine (*Pinus taeda* L.) and their association with fusiform rust gall development. Plant J 51: 1077–1098. 17635765 10.1111/j.1365-313X.2007.03208.x

[86] Johnson C, Kasprzewska A, Tennessen K, Fernandes J, Nan GL, et al (2009) Clusters and superclusters of phased small RNAs in the developing inflorescence of rice. Genome Res 19: 1429–1440. 19584097 10.1101/gr.089854.108 PMC2720183

[87] Li T, Li H, Zhang YX, Liu JY (2011) Identification and analysis of seven HO-responsive miRNAs and 32 new miRNAs in the seedlings of rice (*Oryza sativa* L. ssp. indica). Nucleic Acids Res 39: 2821–2833. 21113019 10.1093/nar/gkq1047 PMC3074118

[88] Addo-Quaye C, Snyder JA, Park YB, Li YF, Sunkar R, et al (2009) Sliced microRNA targets and precise loop-first processing of MIR319 hairpins revealed by analysis of the *Physcomitrella patens* degradome. RNA 15: 2112–2121. 19850910 10.1261/rna.1774909 PMC2779683

[89] Bologna NG, Mateos JL, Bresso EG, Palatnik JF (2009) A loop-to-base processing mechanism underlies the biogenesis of plant microRNAs miR319 and miR159. EMBO J 28: 3646–3656. 19816405 10.1038/emboj.2009.292 PMC2790483

[90] Bender W (2008) MicroRNAs in the *Drosophila* bithorax complex. Genes Dev 22: 14–19. 18172161 10.1101/gad.1614208 PMC2151010

[91] Stark A, Bushati N, Jan CH, Kheradpour P, Hodges E, et al (2008) A single *Hox* locus in *Drosophila* produces functional microRNAs from opposite DNA strands. Genes Dev 22: 8–13. 18172160 10.1101/gad.1613108 PMC2151017

[92] Tyler DM, Okamura K, Chung WJ, Hagen JW, Berezikov E, et al (2008) Functionally distinct regulatory RNAs generated by bidirectional transcription and processing of microRNA loci. Genes Dev 22: 26–36. 18172163 10.1101/gad.1615208 PMC2151012

[93] Rhoades MW, Reinhart BJ, Lim LP, Burge CB, Bartel B, et al (2002) Prediction of plant microRNA targets. Cell 110: 513–520. 12202040 10.1016/s0092-8674(02)00863-2

[94] Zhou L, Liu Y, Liu Z, Kong D, Duan M, et al (2010) Genome-wide identification and analysis of drought-responsive microRNAs in *Oryza sativa* . J Exp Bot 61: 4157–4168. 20729483 10.1093/jxb/erq237

[95] Dai X, Zhuang Z, Zhao PX (2011) Computational analysis of miRNA targets in plants: current status and challenges. Brief Bioinform 12: 115–121. 20858738 10.1093/bib/bbq065

[96] Zhang Y (2005) miRU: an automated plant miRNA target prediction server. Nucleic Acids Res 33: W701–704. 15980567 10.1093/nar/gki383 PMC1160144

[97] Wu B, Li Y, Yan H, Ma Y, Luo H, et al (2012) Comprehensive transcriptome analysis reveals novel genes involved in cardiac glycoside biosynthesis and mlncRNAs associated with secondary metabolism and stress response in *Digitalis purpurea* . BMC Genomics 13: 15. 22233149 10.1186/1471-2164-13-15 PMC3269984

[98] Harris ED (2002) Cellular transporters for zinc. Nutr Rev 60: 121–124. 12002684 10.1301/00296640260085877

[99] Wang M, Xu Q, Yu J, Yuan M (2010) The putative Arabidopsis zinc transporter ZTP29 is involved in the response to salt stress. Plant Mol Biol 73: 467–479. 20358261 10.1007/s11103-010-9633-4

[100] Lu S, Yang C, Chiang VL (2011) Conservation and diversity of microRNA-associated copper-regulatory networks in *Populus trichocarpa* . J Integr Plant Biol 53: 879–891. 22013976 10.1111/j.1744-7909.2011.01080.x

[101] Shi R, Chiang VL (2005) Facile means for quantifying microRNA expression by real-time PCR. Biotechniques 39: 519–525. 16235564 10.2144/000112010

[102] Baskerville S, Bartel DP (2005) Microarray profiling of microRNAs reveals frequent coexpression with neighboring miRNAs and host genes. RNA 11: 241–247. 15701730 10.1261/rna.7240905 PMC1370713

[103] Merchan F, Boualem A, Crespi M, Frugier F (2009) Plant polycistronic precursors containing non-homologous microRNAs target transcripts encoding functionally related proteins. Genome Biol 10: R136. 19951405 10.1186/gb-2009-10-12-r136 PMC2812943

[104] Chen CJ, liu Q, Zhang YC, Qu LH, Chen YQ, et al (2011) Genome-wide discovery and analysis of microRNAs and other small RNAs from rice embryogenic callus. RNA Biol 8: 538–547. 21525786 10.4161/rna.8.3.15199

[105] Luo YC, Zhou H, Li Y, Chen JY, Yang JH, et al (2006) Rice embryogenic calli express a unique set of microRNAs, suggesting regulatory roles of microRNAs in plant post-embryogenic development. FEBS Lett 580: 5111–5116. 16959252 10.1016/j.febslet.2006.08.046

[106] Zhang S, Zhou J, Han S, Yang W, Li W, et al (2010) Four abiotic stress-induced miRNA families differentially regulated in the embryogenic and non-embryogenic callus tissues of *Larix leptolepis* . Biochem Biophys Res Commun 398: 355–360. 20599742 10.1016/j.bbrc.2010.06.056

[107] Wu XM, Liu MY, Ge XX, Xu Q, Guo WW (2011) Stage and tissue-specific modulation of ten conserved miRNAs and their targets during somatic embryogenesis of Valencia sweet orange. Planta 233: 495–505. 21103993 10.1007/s00425-010-1312-9

[108] Chen C, Ridzon DA, Broomer AJ, Zhou Z, Lee DH, et al (2005) Real-time quantification of microRNAs by stem-loop RT-PCR. Nucleic Acids Res 33: e179. 16314309 10.1093/nar/gni178 PMC1292995

[109] Varkonyi-Gasic E, Wu R, Wood M, Walton EF, Hellens RP (2007) Protocol: a highly sensitive RT-PCR method for detection and quantification of microRNAs. Plant Methods 3: 12. 17931426 10.1186/1746-4811-3-12 PMC2225395

[110] Luo ZY, Lu QH, Liu SP, Chen XH, Luo JQ, et al (2003) Screening and identification of novel genes involved in biosynthesis of ginsenoside in *Panax ginseng* plant. Acta Biochim Biophys Sin (Shanghai) 35: 554–560. 12796817

[111] Lung B, Zemann A, Madej MJ, Schuelke M, Techritz S, et al (2006) Identification of small non-coding RNAs from mitochondria and chloroplasts. Nucleic Acids Res 34: 3842–3852. 16899451 10.1093/nar/gkl448 PMC1557801

[112] Gonzalez-Ibeas D, Blanca J, Donaire L, Saladie M, Mascarell-Creus A, et al (2011) Analysis of the melon (*Cucumis melo*) small RNAome by high-throughput pyrosequencing. BMC Genomics 12: 393. 21812964 10.1186/1471-2164-12-393 PMC3163571

[113] Arumuganathan K, Earle ED (1991) Nuclear DNA content of some important plant species. Plant Mol Biol Rep 9: 208–218.

[114] Hong CP, Lee SJ, Park JY, Plaha P, Park YS, et al (2004) Construction of a BAC library of Korean ginseng and initial analysis of BAC-end sequences. Mol Genet Genomics 271: 709–716. 15197578 10.1007/s00438-004-1021-9

[115] Hamilton A, Voinnet O, Chappell L, Baulcombe D (2002) Two classes of short interfering RNA in RNA silencing. EMBO J 21: 4671–4679. 12198169 10.1093/emboj/cdf464 PMC125409

[116] Kasschau KD, Fahlgren N, Chapman EJ, Sullivan CM, Cumbie JS, et al (2007) Genome-wide profiling and analysis of *Arabidopsis* siRNAs. PLoS Biol 5: e57. 17298187 10.1371/journal.pbio.0050057 PMC1820830

[117] Lippman Z, Martienssen R (2004) The role of RNA interference in heterochromatic silencing. Nature 431: 364–370. 15372044 10.1038/nature02875

[118] Aravin AA, Lagos-Quintana M, Yalcin A, Zavolan M, Marks D, et al (2003) The small RNA profile during *Drosophila melanogaster* development. Dev Cell 5: 337–350. 12919683 10.1016/s1534-5807(03)00228-4

[119] Chan SW, Zilberman D, Xie Z, Johansen LK, Carrington JC, et al (2004) RNA silencing genes control de novo DNA methylation. Science 303: 1336. 14988555 10.1126/science.1095989

[120] Zilberman D, Cao X, Jacobsen SE (2003) ARGONAUTE4 control of locus-specific siRNA accumulation and DNA and histone methylation. Science 299: 716–719. 12522258 10.1126/science.1079695

[121] Abdel-Ghany SE, Pilon M (2008) MicroRNA-mediated systemic down-regulation of copper protein expression in response to low copper availability in *Arabidopsis* . J Biol Chem 283: 15932–15945. 18408011 10.1074/jbc.M801406200 PMC3259626

[122] Chen L, Zhang Y, Ren Y, Xu J, Zhang Z, et al (2012) Genome-wide identification of cold-responsive and new microRNAs in Populus tomentosa by high-throughput sequencing. Biochem Biophys Res Commun 417: 892–896. 22209794 10.1016/j.bbrc.2011.12.070

[123] Chiou TJ, Aung K, Lin SI, Wu CC, Chiang SF, et al (2006) Regulation of phosphate homeostasis by microRNA in *Arabidopsis* . Plant Cell 18: 412–421. 16387831 10.1105/tpc.105.038943 PMC1356548

[124] Fujii H, Chiou TJ, Lin SI, Aung K, Zhu JK (2005) A miRNA involved in phosphate-starvation response in *Arabidopsis* . Curr Biol 15: 2038–2043. 16303564 10.1016/j.cub.2005.10.016

[125] Gao P, Bai X, Yang L, Lv D, Pan X, et al (2011) Osa-MIR393: a salinity- and alkaline stress-related microRNA gene. Mol Biol Rep 38: 237–242. 20336383 10.1007/s11033-010-0100-8

[126] Jones-Rhoades MW, Bartel DP (2004) Computational identification of plant microRNAs and their targets, including a stress-induced miRNA. Mol Cell 14: 787–799. 15200956 10.1016/j.molcel.2004.05.027

[127] Sunkar R, Kapoor A, Zhu JK (2006) Posttranscriptional induction of two Cu/Zn superoxide dismutase genes in *Arabidopsis* is mediated by downregulation of miR398 and important for oxidative stress tolerance. Plant Cell 18: 2051–2065. 16861386 10.1105/tpc.106.041673 PMC1533975

[128] Trindade I, Capitao C, Dalmay T, Fevereiro MP, Santos DM (2010) miR398 and miR408 are up-regulated in response to water deficit in *Medicago truncatula* . Planta 231: 705–716. 20012085 10.1007/s00425-009-1078-0

[129] Yamasaki H, Abdel-Ghany SE, Cohu CM, Kobayashi Y, Shikanai T, et al (2007) Regulation of copper homeostasis by micro-RNA in *Arabidopsis* . J Biol Chem 282: 16369–16378. 17405879 10.1074/jbc.M700138200

[130] Zhao B, Ge L, Liang R, Li W, Ruan K, et al (2009) Members of miR-169 family are induced by high salinity and transiently inhibit the NF-YA transcription factor. BMC Mol Biol 10: 29. 19351418 10.1186/1471-2199-10-29 PMC2670843

[131] Zhao B, Liang R, Ge L, Li W, Xiao H, et al (2007) Identification of drought-induced microRNAs in rice. Biochem Biophys Res Commun 354: 585–590. 17254555 10.1016/j.bbrc.2007.01.022

[132] Zhou X, Wang G, Zhang W (2007) UV-B responsive microRNA genes in *Arabidopsis thaliana* . Mol Syst Biol 3: 103. 17437028 10.1038/msb4100143 PMC1865585

[133] Choi YE (2006) Ginseng (*Panax ginseng*). Methods Mol Biol 344: 361–371. 17033078 10.1385/1-59745-131-2:361

[134] Hafner M, Landgraf P, Ludwig J, Rice A, Ojo T, et al (2008) Identification of microRNAs and other small regulatory RNAs using cDNA library sequencing. Methods 44: 3–12. 18158127 10.1016/j.ymeth.2007.09.009 PMC2847350

[135] Masoudi-Nejad A, Tonomura K, Kawashima S, Moriya Y, Suzuki M, et al (2006) EGassembler: online bioinformatics service for large-scale processing, clustering and assembling ESTs and genomic DNA fragments. Nucleic Acids Res 34: W459–462. 16845049 10.1093/nar/gkl066 PMC1538775

[136] Ewing B, Green P (1998) Base-calling of automated sequencer traces using phred. II. Error probabilities. Genome Res 8: 186–194. 9521922

[137] Meyers BC, Axtell MJ, Bartel B, Bartel DP, Baulcombe D, et al (2008) Criteria for annotation of plant microRNAs. Plant Cell 20: 3186–3190. 19074682 10.1105/tpc.108.064311 PMC2630443

[138] Zhang BH, Pan XP, Cox SB, Cobb GP, Anderson TA (2006) Evidence that miRNAs are different from other RNAs. Cell Mol Life Sci 63: 246–254. 16395542 10.1007/s00018-005-5467-7 PMC11136112

[139] Livak KJ, Schmittgen TD (2001) Analysis of relative gene expression data using real-time quantitative PCR and the 2(-Delta Delta C(T)) Method. Methods 25: 402–408. 11846609 10.1006/meth.2001.1262

