# Supplementary material for: High-Throughput Sequencing and Characterization of the Small RNA Transcriptome Reveal Features of Novel and Conserved MicroRNAs in Panax ginseng
Source: PLoS One. 2012 Sep 4;7(9):e44385. doi: 10.1371/journal.pone.0044385 (PMC3433442; doi:10.1371/journal.pone.0044385)
Supplement: Figure S1 — Predicted hairpin structures of P. ginseng miRNA precursors. Mature miRNA sequences are indicated in red. miRNA* sequences are indicated in blue. (DOC) [file pone.0044385.s001.doc]

**pgi-MIR482a (dG=-48.4)**

AUG U CUUA- ACU

5’-GAG GGUCUUAUGAAGGCAGAGG AGUGGGAGG GCAAGAAAAAUAAACA U

||| | ||||||||| || || ||||||||| |||||||||||| ||

3’-CUC UUGGAGUACUUUGGUAACC UUACCCUCC CGUUCUUUUUGUCGGU C

A-- - UUAAC CAA

**pgi-MIR482b (dG=-59.5)**

CU G- A-- A AUA AACCC CCCC

5’-AUUUUUCUUGCCAAUUC CCCAUUCCAAUGGUUUCAU AGGUU CUCCUUUUCCU UC UCCUUGU AACAC A

||||||||| |||| || ||||||| ||| |||||| ||||| ||||||||||| || || |||| |||||

3’-UGAAAAGAAAGGUUUAG GGGUAGGUUUGUUGAAGUA UCUAG GAGGGAAAGGG AG AGAGACA UUGUG /

U- GA AUA - AG- GC--- CAAU

**pgi-MIR2118 (dG=-62.6)**

U U-- AUA UUU AUUU- AAG GU-- A

5’-GAGAGGAG AAG GGGAAAGGGAG GAUCUAGAUGAAGUUG GGAUGGGUG GGAAAGAA UUUU GU G

|| ||||| ||| ||||| ||||| || ||||| ||||||| ||||||||| |||||||| |||| ||

3’-UUGUCCUU UUC CCCUUCCCCUC CUCGGUCUCCUUUAGU CCUACCCAC CCUUUCUU AGAA CA A

- UUU --- UAC CUUAU AA- AGUU U

**pgi-MIR4376 (dG=-71.2)**

G AAAG GA AA AGCU

5’-AGCAUGG AAGGUUUUCUAUGCAGGAGAGAUGACGCCCAUCCGUACGC CAA UCAAUUAUCCUCUU UCACC G

||||||| |||||||| |||||||| |||||| ||||| |||| ||| ||| ||||||| |||||| || ||

3’-UCGUAUU UUCUAAAACAUACGUCCCCUUUACCGCGGGCGGGUACGUG GUU AGUUAAUUGGAGAG AGAGG U

- ---- G- -- AAUG

**pgi-MIR6135a (dG=-58.1)**

AUU - C AA

5’-AUGAAGUU UUUUUGG UAAGUUGGUCAAUUGGC AAUAAAAUACUGACACACAUAC C

|||||||| ||||||| ||||||||||||||||| |||| |||||| |||| || ||

3’-UACUUCAA AAAAACC AUUCAACCAGUUAACUG UUAUCUUAUGAGUGUGCGUCUG U

AU- U - CU

**pgi-MIR6135b (dG=-68.2)**

- UUU AG

5’-GUUAGCAUGAAG UUAUUUU GGUAAGUUGGUCAAUUGGCCAAUAGAAUGCUGACACACAUAC U

|||||||||||| ||||||| ||||||||||||||||||| ||||||||| |||||| || ||

3’-CAAUCGUAUUUC AAUAAAA UCAUUCAAUCAGUUAACCGAUUAUCUUAUAAUUGUGCGUCUG C

A CC- CU

**pgi-MIR6135c (dG=-50.7)**

AU GGCGA

5’-AAGUUU UUUUGGGUAAGUUGGUCAAUUGACCAAUAGAGUAUUAACAUGUA G

|||||| |||||||||||||||||||||| || ||||||||||||| ||

3’-UUCAAA AAAACCCAUUCAACUAGUUAACCAGUCAUUUUAUAAUUGUGUGU A

-- AUUUC

**pgi-MIR6135d (dG=-72.9)**

UA- GGUGA

5’-GGUUAGUAUGAAGUU UUUUUGGGUAAGUUGGUCAAUUGACCAAUAGAAUAAUGACACGCA G

||||||||||||||| ||||||||||||||||| ||||| ||||||||||| |||||||||

3’-CCAAUCGUACUUCAA AAAAAUUCAUUCAACUAAUUAACCGGUUAUCUUAUGACUGUGUGU G

UAA AUGUU

miR6135e.1

miR6135e.2

**pgi-MIR6135e (dG=-82.2)**

U GG- - A A GGCGA

5’-AUGGGAAGUGGUUAGCAUGAAG UUAUUUU GUAAGUUGGUCAAUUGA CUAAUAGA UACUG CACGCA G

|||||| |||||||||||||| ||||||| ||||||||||||||||| |||||||| ||||| ||||||

3’-UACCCUAGGCCAAUCGUACUUC AAUAAAA CAUUCAACCAGUUAACU GGUUAUUU AUGAU GUGUGU /

- AAA C - - AUCUU

**pgi-MIR6135f (dG=-59.6)**

ACAUAGAG

5’-AUGAAUUUUAUUUUUGAGUAAGUUGGUCAAUUGGCUAAUAGAAUGCUGACAC C

||||| ||||||||||||||||||||||| ||| ||||||| |||||||||

3’-UACUUCAAGUAAAAACUCAUUUAAUCAGUAAACUAGUUAUCUCACGACUGUG U

CGACCGCU

**pgi-MIR6135g (dG=-57.1)**

CG

5’-GUUUAUUUUUGAGUAAGUUGGUCAAUUGGCAAAUAGAAUAUUGACACGCAGG A

||||||||||||||||||||||||||||| |||||||| |||| |||||||

3’-CAAAUAAAAACUCAUUUAAUCAGUUAACCAGUUAUCUUACGAUUAUGUGUCU A

UG

**pgi-MIR6135h (dG=-42.0)**

CACGCAAGCGA

5’-GUAUUAAGUUCAUUUUUACGUAAGUUGGUCAAUUGGCCAAUAGAGUGCUGA G

|||| ||||| |||||| |||||||||||||||| |||||||||| |||

3’-CGUACUUCAAAUAAAAAACUAUUUAACCAGUUAACUAGUUAUCUUAUAACU A

AUAUGUAUCUC

**pgi-MIR6135i (dG=-62.9)** (miR6135d-antisense)

AUU UACAA

5’-GGUUAGCAUGAAGUU UUUUUAAGUAAGUUGAUUAAUUGGCCAAUAGAAUACUGACACACA C

|||||| |||||||| ||||| |||||||| ||||||||||||||||||| |||||| ||

3’-CCAAUCAUACUUCAA AAAAACCCAUUCAACCAGUUAACUGGUUAUCUUAUUACUGUGCGU C

AU- CCACU

**pgi-MIR6135j (dG=-62.2)**

U GG- AUC A

5’-GGAAGCGGUUAGUAUGAAGUU AUUUU GGUAAGUUG AAUUGACUAAUAGAAUACUGACACGCAGGCG G

||| |||||||||||||||| ||||| ||||||||| |||||| |||||||||||||| ||||| |||

3’-CCUAGGUCAAUUAUACUUCAA UAAAA CUAUUUAAU UUAACUAGUUAUUUUAUGACUAUGCGUAUGU G

- AAA CAA U

**pgi-MIR6135k (dG=-80.1)**

CAUGAUAAGU A

5’-UACGGAUCUGGUUAUUAUGAAGUUAAUUU UGGUCAAUUGACCAAUAUAGUGUUGACAUGCAGACAAAGAG U

|||||| |||||| |||||||||||||| |||| ||||||||||||||||||||||||||||||| ||||

3’-GUGCCUUUACUAAUCGUACUUCAAUUGAA ACUAAUUAAUUGGUUAUGUCAUAGCUGUGCGUCUGUCUUUC U

---------- A

**pgi-MIR6136a (dG=-109.2)**

A- CA-- G - CUAUA

5’-UAUAU CUUAUUUUACUCACCCGUGCU UCUCUUUAGACGACG UUUCGGUCAUACAACCAUCAUUUAUACUUUUAG CGACG U

||||| ||||||||||||||||||||| ||||||||| ||||| |||||||||||||||| || ||||||||||||| |||||

3’-GUAUG GAAUAGAAUGAGUGGGCACGA AGGGAAAUCCGCUGC AAAGCCAGUAUGUUGGCAGCAGAUAUGAAAAUC GCUGC G

GG UUGG - A CUGUU

miR6136a.1

miR6136a.2

**pgi-MIR6136b (dG=-107.4)** (miR6136a-antisense)

AACC - U GACAA

5’-CUUAUCUUACUCACCCGUGCU UCCCUUUAGGCGACG UUUCGGUCAUACAACCGUCGUCUAUACUUUUAG CGACG C

||||| ||||||||||||||| || |||||||||||| ||||||||||||||||||||| ||||||||||| |||||

3’-GAAUAAAAUGAGUGGGCACGA AGAGAAAUCUGCUGC AAAGCCAGUAUGUUGGUAGUAAAUAUGAAAAUC GCUGC A

GU-- C - GAUAU

**pgi-MIR6137a (dG=-70.4)**

U AGA

5’- UUUCACAUGACUUUUUGAUAUUUUCACAUGAAAAUUGUCGCUAUAGAUCCUAUC U

|||||||| ||||||||||||||||||||||||| |||| ||||||||||||||

3’- AAAGUGUAAUGAAAAACUAUAAAGGUGUACUUUUCACAGAGAUAUCUAGGAUAG /

A AUA

**pgi-MIR6137b (dG=-56.1)**

CC G

5’-AUAUUUUCACAUGAAAAUUGUCGCUAUAGAU UAUAGCGAUGGUUAUAGC A

|||||| |||||||||| ||||||||||||| ||||||||| |||| |||

3’-UAUAAAUGUGUACUUUUCACAGCGAUAUCUA AUAUCGCUGGCAAUUUUG C

C- A

**pgi-MIR6138 (dG=-79.1)**

AU-- GAU------- CA - AC CCC AAA

5’-GUUACGUUUGGAUUGAAGGAAUGAA GGAAUGGAAUGAAGAGAUGAAU UUGUUCCAUUUCAGU GAAUGAGUCAUUC CAU CCU CCAAGUAAU A

||||||||||||||| ||| |||| |||||||||||| ||| |||| |||||| ||| ||| ||||||||||||| ||| ||| |||||| ||

3’-CGAUGCAAACCUAGCCACCUCACUU CCUUACCUUACUCUUUUUUUUA AACAAGAUAACCUUA CUUACUUAGUAAG GUA GGA GGUUUAAUA /

GCUC AACACAAACC C- A A- A-- CUU

**pgi-MIR6139 (dG=-38.0)**

ACCAA U CAAUUACUU UCAA UUA

5’- UUUAUUC CUUUCUCAAUGUUUCUUAUUCAAUUCAAU UUUAU CCAA C

||| ||| ||||||||||| ||||||||||||||||| ||||| ||||

3’- AAAGAAG GGAAGGGUUACUAAGAAUAAGUUAAGUUA AGAUA GGUU /

UUUCG - --------- UUA- CAU

**pgi-MIR6140a (dG=-78.7)**

CAU CAU AUCAACAAAUUGCUUCACU

5’-GAGUAAUGCUA ACACAAACUAUUCUACAAACAUUUUACAAAUUGUUGAUGUGACAUACUCCA CAACAUAUUG G

||| ||||||| |||||||||||||||||||||||||| |||||||||||||| |||| |||| || |||||||

3’-CUUUUUACGAU UGUGUUUGAUAAGAUGUUUGUAAAAUAUUUGACGACUACACCGUAUAAGGU GUAGUAUAAC U

CUC AGC ACACCCCGGGCUUUCUGAU

**pgi-MIR6140b (dG=-75.2)**

U U GAG UUACAAA - UUUUUAUGUGGGGCCAAAGACUAC

5’- UUAAA GAAUAAUGCUA ACACAAACUAUUCUGCAAAUAUU UUGCUGAUGUGGCAUUCUCCA CCUCAGC A

||||| || |||||||| ||||||| ||||||||||||||| ||||||||||||||| || || |||||||

3’- AAUUU CUCAUUACGAU UGUGUUUAAUAAGAUGUUUGUAA AAUGACUACACUGUAUGAUGU GGAGUUG /

A - GGA ------- G UAUAACUAGUUGUUUAACAACGUU

**pgi-MIR6140c (dG=-69.8)**

GAC ACAUAU ACCGAA UAU A

5’-ACACAAACUAUUU AAACAUUUUACAAACUGCUGAUGUAGCAUAUUCCACUUCA UGUGUUGG GAC AUUG A

||||||||||||| ||| |||||| ||||||||||||| ||||||||||||||| |||||||| ||| ||||

3’-UGUGUUUGAUAAA UUUAUAAAAUAUUUGACGACUACACCGUAUGAGGUGGAGU GCAUAACU UUG UAAC /

AUA C----- AG---- UU- A

**pgi-MIR6140d (dG=-44.9)**

C UGUGGGGUUGAAAACUAC

5’- AAAUCGUUGAUGUGGCAUACUUCACCUCAUCAUAUUG A

|||| |||||||||||||| ||||||||| ||||||

3’- UUUGACGACUACACCGUAUAAGGUGGAGUUAUAUAAC /

A UAAUUGUUUAACAAAGUU

**pgi-MIR6141 (dG=-196.5)**

UUC A- C A AAGGAAUUUUUUCCA ---- GAAU

5’-AAUUCUCUCGGAACAA CGAAUUUACAACUCAUCAAUUG UAAUUCAAUGGUUCUGACUCCCAUUGCGUG GAGCCCAAUU ACUAAAUCUGGCCUGUAGCGGACCAGCUUA UUUUUGGAUCUGUGAUUGAUCCUACGGAU UCUA U

|||||||||||||||| |||||||||||||||||||||| |||||||||||||||| ||||||||||||| ||| |||||| ||||||| ||||| ||| |||||||||||| |||||||||||||||||||| |||||||| ||||

3’-UUAAGAGAGCCUUGUU GCUUAAAUGUUGAGUAGUUAAC GUUAGGUUACCAAGACCGAGGGUAAUGCAC CUCUGGUUAA UGAUUUAUACUGGUUAUNGCCUGGUCGGAU AAAAACUUAGACACUAACUAUGAUGCCUA GGAU U

UAA CA - - CUUCGGUA------- AUAU AUCU

**pgi-MIR6142 (dG=-67.0)**

CGA- C- AC U AUUCUAGAUC

5’-GUACACAGU CUAUGAAGA GAUUUUUUGGGCUAUG GACGG UUCAACUGUAGUCAUAGCU U

||||||||| ||||| || ||||||||| |||||| ||||| |||| |||| |||||||||

3’-UAUGUGUCA GAUACCGCU CUAAAAAACGCGAUAC CUGCC AAGUGGGUAGCAGUAUCGG G

ACCC AA C- - GCGGCAUCGG

**pgi-MIR6143a (dG=-49.8)**

- GA

5’-UUUAUUUCAUGUCCAAUACAGUGUUGACAUACAGUCG AGAG U

||||||||||||||||||||||||||| | ||| || ||||

3’-AAGUGAAGUACGGGUUAUGUCAUGACUAUGCGUCCGC UCUC G

C UA

miR6143b-5p

**pgi-MIR6143b (dG=-72.0)**

GAUAAA AAGUUUAU G AU

5’-GGUUAGCAUGAA GUGAACUUCAUAGUAGU UUCAUGAUUAAUACAAUGUCGACACGCAGGCG AGAG C

|||||||||||| ||||| ||||| ||||| |||||| |||||||| ||| |||||||||||| ||||

3’-CCAGUCGUACUU UACUUUAAGUAGUAUUA AAGUACAAGUUAUGUCACGACUGUGUGUCCGC UCUC A

------ -------- - CU

miR6143b-3p

**Figure S1.** Predicted hairpin structures of *P. ginseng* miRNA precursors. Mature miRNA sequences are indicated in red. miRNA* sequences are indicated in blue.
