# Supplementary material for: High-Throughput Sequencing and Characterization of the Small RNA Transcriptome Reveal Features of Novel and Conserved MicroRNAs in Panax ginseng
Source: PLoS One. 2012 Sep 4;7(9):e44385. doi: 10.1371/journal.pone.0044385 (PMC3433442; doi:10.1371/journal.pone.0044385)
Supplement: Figure S2 — The quality and quantity of total RNA used for small RNA library construction. One µl of total RNA were diluted with 1 µl of RNase-free water and then denatured at 70°C for two min. Total RNA was analyzed using Agilent 2100. The results showed that the sample had a RNA integrity number (RIN) of 8.1 and a 28s/18s rRNA ratio of 1.8, suggesting the RNA samples were not degraded. The concentration of original total RNA is 302 ng/µl, which is good for small RNA library construction. (DOC) [file pone.0044385.s002.doc]

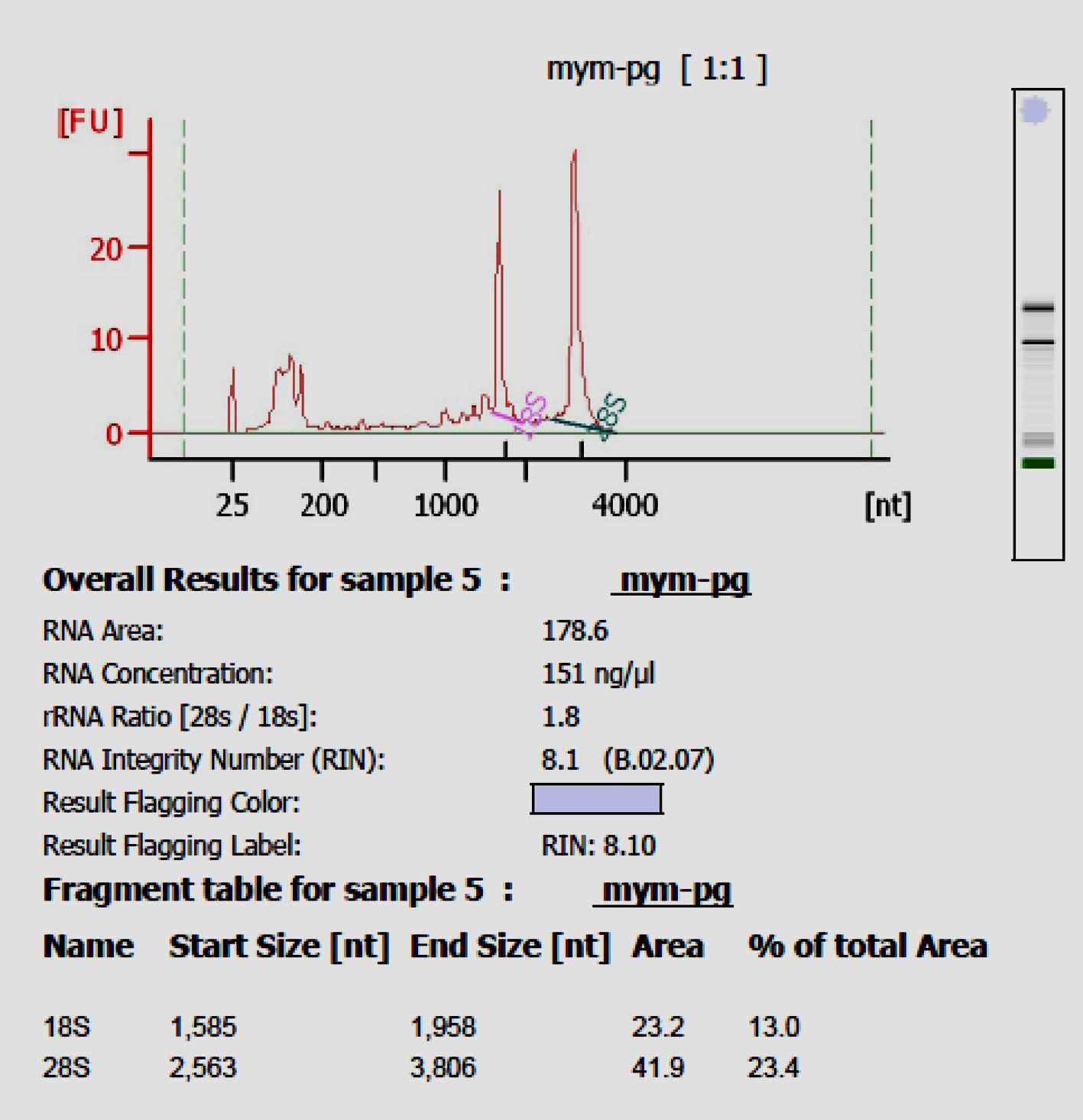


**Figure S2.** The quality and quantity of total RNA used for small RNA library construction. One µl of total RNA were diluted with 1 µl of RNase-free water and then denatured at 70 °C for two min. Total RNA was analyzed using Agilent 2100. The results showed that the sample had a RNA integrity number (RIN) of 8.1 and a 28s/18s rRNA ratio of 1.8, suggesting the RNA samples were not degraded. The concentration of original total RNA is 302 ng/µl, which is good for small RNA library construction.
