# Supplementary material for: High-Throughput Sequencing and Characterization of the Small RNA Transcriptome Reveal Features of Novel and Conserved MicroRNAs in Panax ginseng
Source: PLoS One. 2012 Sep 4;7(9):e44385. doi: 10.1371/journal.pone.0044385 (PMC3433442; doi:10.1371/journal.pone.0044385)
Supplement: Table S1 — Statistics of high-throughput sequencing results of P. ginseng small RNAs. (DOC) [file pone.0044385.s003.doc]

**Table S1.** Statistics of high-throughput sequencing results of *P. ginseng* small RNAs.

|  | **Number** | **Percentage (%)** |
| --- | --- | --- |
| **Total Reads** | 13326328 | 100.0 |
| **Low Quality** | 617363 | 4.6 |
| **High Quality** | 12629519 | 94.8 |
| **adaptor3 null** | 12877 | 0.1 |
| **insert null** | 143808 | 1.1 |
| **5' adaptor contaminants** | 3234 | 0.0 |
| **size < 18 nt** | 468573 | 3.5 |
| **polyA** | 436 | 0.0 |
| **size >= 18 nt** | 12000591 | 90.1 |
