# Supplementary material for: High-Throughput Sequencing and Characterization of the Small RNA Transcriptome Reveal Features of Novel and Conserved MicroRNAs in Panax ginseng
Source: PLoS One. 2012 Sep 4;7(9):e44385. doi: 10.1371/journal.pone.0044385 (PMC3433442; doi:10.1371/journal.pone.0044385)
Supplement: Table S2 — Conserved miRNAs in P. ginseng . (DOC) [file pone.0044385.s004.doc]

**Table S2.** Conserved miRNAs in *P. ginseng*.

| **miRNA family** | **total reads** | **miRNA name** | **miRNA sequence** **(5'→3')** |
| --- | --- | --- | --- |
| MIR156 | 166 | miR156a | TTGACAGAAGATAGAGAGCAC |
|  |  | miR156b | TTGACAGAAGAGAGGGAGCAC |
| MIR159 | 61264 | miR159a | TTTGGATTGAAGGGAGCTCTA |
|  |  | miR159b | TTTGGATTGAAGGGAGCTCTT |
|  |  | miR159c | TTTGGATTGAAGGGAGCTCTC |
|  |  | miR159d | TTTGGATTGAAGGGAGCTCTG |
|  |  | miR159e | TCTGGATTGAAGGGAGCTCTA |
|  |  | miR159f | TATGGATTGAAGGGAGCTCTA |
|  |  | miR159g | CTTGGATTGAAGGGAGCTCTA |
| MIR160 | 94 | miR160a | TGCCTGGCTCCCTGTATGC |
|  |  | miR160b | TGCCTGGCTCCCTGCATGC |
| MIR162 | 48 | miR162 | TCGATAAACCTCTGCATCCAG |
| MIR164 | 1 | miR164 | TGGAGAAGCAGGGCACGTGCA |
| MIR166 | 228538 | miR166a | TCGGACCAGGCTTCATTCCTC |
|  |  | miR166b | TCGGATCAGGCTTCATTCCTC |
|  |  | miR166c | TCGGACCAGGCTTCATTCCTT |
|  |  | miR166d | TCGGACCAGGCTTCATTCCTG |
|  |  | miR166e | TCGGACCAGGCTTCATTCCTA |
|  |  | miR166f | TCGGACCAGGCTTCATTCCCC |
| MIR167 | 2044 | miR167a | TGAAGCTGCCAGCATGATCTG |
|  |  | miR167b | TGAAGCTGCCAGCATGATCTAA |
|  |  | miR167c | TGAAGCTGCCAGCATGATCTA |
|  |  | miR167d | TGAAGCTGCCAGCATGATCTGA |
|  |  | miR167e | GAAGCTGCCAGCATGATCTGG |
|  |  | miR167f | TGAAGCTGCCAGCATGATCTAT |
|  |  | miR167g | TGAAGCTGCCAGCATGATCTT |
|  |  | miR167h | TGAAGCTGCCAGCATGATCTAG |
| MIR168 | 36 | miR168a | TCGCTTGGTGCAGGTCGGGA |
|  |  | miR168b | TCGCTTGGTGCAGATCGGGAC |
|  |  | miR168c | TCGCTTGGTGCAGGTCGGGAA |
| MIR169 | 3 | miR169a | CAGCCAAGGATGACTTGCCGA |
|  |  | miR169b | CAGCCAAGGATGACTTGCCGG |
| MIR171 | 12 | miR171a | TTGAGCCGTGCCAATATCACG |
|  |  | miR171b | TTGAGCTGTGCCAATATCACG |
| MIR172 | 332 | miR172a | AGAATCTTGATGATGCTGCAT |
|  |  | miR172b | AGAATCTTGATGATGCTGCAG |
|  |  | miR172c | ACAATCTTGATGATGCTGCAT |
|  |  | miR172d | AGAATTTTGATGATGCTGCAT |
| MIR319 | 586 | miR319 | TTGGACTGAAGGGAGCTCCC |
| MIR390 | 3 | miR390 | AAGCTCAGGAGGGATAGCGCC |
| MIR393 | 301 | miR393 | TCCAAAGGGATCGCATTGATC |
| MIR394 | 2 | miR394 | TTGGCATTCTGTCCACCTCC |
| MIR395 | 3 | miR395 | CTGAAGTGTTTGGGGGAACTC |
| MIR396 | 4021 | miR396a | TTCCACGGCTTTCTTGAACTG |
|  |  | miR396b | TTCCACAGCTTTCTTGAACTT |
|  |  | miR396c | TTCCACAGCTTTCTTGAACTG |
|  |  | miR396d | TCCCACGGCTTTCTTGAACTG |
|  |  | miR396e | TTCCACGGCTTTCTTGAACTT |
|  |  | miR396f | TTTCACGGCTTTCTTGAACTG |
| MIR397 | 11 | miR397 | ATTGAGTGCAGCGTTGATGAA |
| MIR399 | 28 | miR399 | TGCCAAAGGAGATTTGCCCGG |
| MIR403 | 2730 | miR403 | TTAGATTCACGCACAAACTCG |
| MIR482 | 4 | miR482 | TCTTGCCAATTCCTCCCATTCC |
| MIR827 | 2 | miR827 | TTAGATGACCATCAGCAAACA |
| MIR894 | 4425 | miR894a | GTTTCACGTCGGGTTCACCA |
|  |  | miR894b | ATTCACGTCGGGTTCACCA |
|  |  | miR894c | GGTTCACGTCGGGTTCACCA |
| MIR1024 | 1 | miR1024 | TCAGTTTGGATTGTAGGC |
| MIR1439 | 3 | miR1439 | CTTATATTAAGGAACGGAGTGAGT |
| MIR1509 | 2 | miR1509a | TTAAACAGGGAAATCACGGTTG |
|  |  | miR1509b | TTAAACAAAGAAATCACGGTTG |
| MIR1510 | 2 | miR1510 | GTTGTTTTACCTATTCCACCC |
| MIR1863 | 1 | miR1863 | ATTGTAACATGGTATCAGAGC |
| MIR2118 | 2 | miR2118 | TTTCCTATTCCACCCATCCCAT |
| MIR4376 | 26 | miR4376 | TGCAGGAGAGATGACGCCCATC |
| MIR5072 | 6 | miR5072a | GATCCCCAGCGGAGTCGCCA |
|  |  | miR5072b | CGTCCCCAGCGGAGTCGCC |
|  |  | miR5072c | TCTCCCCAGCGGAGTCGCCA |
|  |  | miR5072d | CGTTCCCCAGCGGAGTCGCCA |
| MIR5079 | 1 | miR5079 | AATTTTGACCTGTTATTTTGGT |
| MIR5139 | 1046 | miR5139a | CGTAACCTGGCTCTGATACCA |
|  |  | miR5139b | CGGAACCTGGCTCTGATACCA |
|  |  | miR5139c | CGCAACCTGGCTCTGATACCA |
