# Supplementary material for: High-Throughput Sequencing and Characterization of the Small RNA Transcriptome Reveal Features of Novel and Conserved MicroRNAs in Panax ginseng
Source: PLoS One. 2012 Sep 4;7(9):e44385. doi: 10.1371/journal.pone.0044385 (PMC3433442; doi:10.1371/journal.pone.0044385)
Supplement: Table S3 — miRNAs* putatively produced from conserved miRNA gene families in P. ginseng . (DOC) [file pone.0044385.s005.doc]

**Table S3.** miRNAs* putatively produced from conserved miRNA gene families in *P. ginseng*.

| **miRNA family** | **total reads** | **miRNA* sequence** **(5'→3')** |
| --- | --- | --- |
| MIR156 | 96 | GCTCTCTAGTCTTCTGTCATC |
|  |  | GCCCTAGTCTTCTGTCATC |
|  |  | GCTCACCCTCTATCTGTCAC |
|  |  | GCTCACTCTCTATCTGTCACC |
|  |  | GCTTACTCTCTATCTGTCACC |
| MIR166 | 209 | GGAATGTTGTCTGGCTCGAGG |
|  |  | GAATGTTGTCTGGCTCGAGGA |
|  |  | GGAATGTTGTCTGGCTCGAGGC |
|  |  | GGAATGTTGTCTGGCTCGAGGT |
|  |  | GAATGCTGTCTGGTTCGAGA |
|  |  | AATGAAGTTTGATCCAAGATC |
| MIR167 | 5 | GATCATGTGGTAGCTTCACC |
|  |  | GATCATGTGGTAGTTTCACC |
|  |  | AGATCATGTGGTAGTTTCAC |
| MIR171 | 1 | TGTTGGAATGGCTCAATCAAA |
| MIR172 | 2 | GTAGCATCATCAAGATTCAC |
|  |  | GTGGCACCATCAAGATTC |
| MIR390 | 2 | GCTATCCATCCTGAGTTTCA |
|  |  | GCTATCTATCCTGAGTTTCA |
| MIR396 | 86 | GTTCAATAAAGCTGTGGGAAG |
| MIR482 | 1  1 | GGGTGTTGGAGAGTAGGA  TAGTGGGAGGCTTAGCAAGAAA^a^ |
| MIR482/ MIR2118 | 1 | TGGATGGGTGATTTGGAAAG^a^ |
| MIR4376 | 4 | CGGGCGCCATTTCCCCTGCATA^a^ |

^a^ The miRNA* sequences were identified by mapping the small RNAs to miRNA precursors identified from the unigene set of *P. ginseng*.
