# Supplementary material for: High-Throughput Sequencing and Characterization of the Small RNA Transcriptome Reveal Features of Novel and Conserved MicroRNAs in Panax ginseng
Source: PLoS One. 2012 Sep 4;7(9):e44385. doi: 10.1371/journal.pone.0044385 (PMC3433442; doi:10.1371/journal.pone.0044385)
Supplement: Table S4 — Targets of conserved miRNAs in P. ginseng . (DOC) [file pone.0044385.s006.doc]

**Table S4. Targets of conserved miRNAs in *P. ginseng*.**

| **miRNA family** | **Target function** | **Unigene ID (penalty score)** |
| --- | --- | --- |
| MIR156 | Squamosa promoter-binding-like protein (SPL) | FW1NBNE01AR7A8(0)^a^, PUT-183a-Panax_ginseng-3299(05) ^a^, FW1NBNE01BZYCE(1),FW1NBNE01BWUZ2(1.5) , PUT-183a-Panax_ginseng-17808(2.5), FW1NBNE01AJW98(3) |
|  | Protein phosphatase 2c | PUT-183a-Panax_ginseng-12734(3) |
|  | Small nuclear ribonucleoprotein | PUT-183a-Panax_ginseng-3569(3) |
| MIR159 | Cytosolic purine 5-nucleotidase | FW1NBNE01B4A7V(2.5) |
|  | Aldehyde dehydrogenase | Contig1475(2.5) |
|  | COP9 signalosome subunit 6 | PUT-183a-Panax_ginseng-7461(2.5) |
|  | WD40 repeat protein | PUT-183a-Panax_ginseng-11636(3) |
|  | Carboxyl-terminal peptidase | PUT-183a-Panax_ginseng-13428(3) |
|  | Copine | PUT-183a-Panax_ginseng-6110(3) |
| MIR162 | Polyamine oxidase | Contig2823(3) |
| MIR164 | NAC domain protein | PUT-183a-Panax_ginseng-12771(1.5) ^a^ |
| MIR166 | Homeobox-leucine zipper protein | PUT-183a-Panax_ginseng-17526(2), PUT-183a-Panax_ginseng-9701(2) |
|  | Histone H1 | PUT-183a-Panax_ginseng-3608(3) |
|  | Calcium-dependent protein kinase | FW1NBNE01A5IBG(3) |
| MIR167 | Auxin response factor (ARF) | PUT-183a-Panax_ginseng-2754(2),PUT-183a-Panax_ginseng-19848(2),   PUT-183a-Panax_ginseng-4258(3),PUT-183a-Panax_ginseng-7927(3) |
|  | Protein disulfide isomerase | PUT-183a-Panax_ginseng-19550(2.5) |
|  | Elongation factor like protein | PUT-183a-Panax_ginseng-4855(3) |
|  | NAC domain-containing protein | FW1NBNE01A4PDR(3) |
|  | 14-3-3 protein | PUT-183a-Panax_ginseng-12182(3) |
| MIR169 | CCAAT-box binding factor | PUT-183a-Panax_ginseng-7042(3) |
|  | Metalloendopeptidase | Contig2946(3) |
| MIR171 | GRAS family transcription factor | PUT-183a-Panax_ginseng-5279(1.5)^a^ , PUT-183a-Panax_ginseng-16749(1  .5) ^a^, PUT-183a-Panax_ginseng-4076(2.5) |
|  | Putative DnaJ protein | PUT-183a-Panax_ginseng-7136(2.5) |
|  | Unknown | Contig1088(3), FW1NBNE01BRMW9(2.5) |
| MIR172 | AP2 domain-containing protein | PUT-183a-Panax_ginseng-1543(1.5) ^a^ |
|  | Dynamin family protein | PUT-183a-Panax_ginseng-18181(2), PUT-183a-Panax_ginseng-4811(3) |
|  | Mitochondrial FAD carrier | PUT-183a-Panax_ginseng-3880(2.5) |
|  | Asparagine synthetase | PUT-183a-Panax_ginseng-14906(3) |
|  | Unknown | FW1NBNE01BOE2O(1.5), FW1NBNE01A893Z(2.5), Contig2556(3) |
| MIR390 | Phytosulfokine receptor | FW1NBNE01CHPEM(3) |
| MIR393 | F-box family protein | Contig1408(1), PUT-183a-Panax_ginseng-4388(3),PUT-183a-Panax_ginseng-10235(2.5) |
| MIR394 | F-box family protein | PUT-183a-Panax_ginseng-18228(1) |
| MIR395 | ATP sulfurylase | Contig1276(1.5) ^a^ |
| MIR396 | Growth-regulating factor (GRF) | FW1NBNE01BIR6Z(2) ^a^ |
|  | Ethylene-overproduction protein | PUT-183a-Panax_ginseng-2170(2.5) |
|  | Cysteine protease | PUT-183a-Panax_ginseng-11588(2.5), PUT-183a-Panax_ginseng-3481(3) |
|  | Leucine carboxyl methyltransferase | PUT-183a-Panax_ginseng-20423(3) |
|  | Chlorophyll synthase | PUT-183a-Panax_ginseng-9327(3) |
|  | Argonaute | Contig1985(3) |
| MIR397 | Laccase | PUT-183a-Panax_ginseng-10167(1) |
|  | Phox domain-containing protein | PUT-183a-Panax_ginseng-1554(2.5) |
|  | CCR4-NOT transcription factor | PUT-183a-Panax_ginseng-4582(3) |
|  | Patellin-4 | FW1NBNE01BXUC4(3) |
|  | R3H domain containing protein | Contig1631(3) |
| MIR399 | Phosphoglycerate mutase | PUT-183a-Panax_ginseng-17927(3) |
|  | Ethylene-overproduction protein | Contig1230(3) |
| MIR403 | Ethylene signaling protein | FW1NBNE01BP8F3(2) |
| MIR482 | TIR-NBS disease resistance protein | FW1NBNE01A3PPX(3) |
|  | Glucosyl/glucuronosyl transferases | Contig1193(2.5) |
|  | Serine endopeptidase | PUT-183a-Panax_ginseng-4272(3) |
|  | Unknown | FW1NBNE01CB4VI(3) |
| MIR827 | DNA binding protein | FW1NBNE01A6XCY(3) |
|  | Unknown | PUT-183a-Panax_ginseng-12977(3), PUT-183a-Panax_ginseng-12972(3) |
| MIR1439 | ZIP transporter | PUT-183a-Panax_ginseng-20083(2) |
|  | Racemase and epimerase | PUT-183a-Panax_ginseng-12684(3) |
|  | Mitogen-activated protein kinase | PUT-183a-Panax_ginseng-7849(3) |
|  | Unknown | FW1NBNE01CFTNQ(3) |
| MIR1509 | Amino acid binding protein | PUT-183a-Panax_ginseng-7580(3) |
|  | Proteolipid subunit of vacuolar H^+^ ATPase | PUT-183a-Panax_ginseng-7089(2.5) |
|  | Galactosyltransferase | FW1NBNE01ANG78(3) |
| MIR1510 | TIR-NBS disease resistance protein | FW1NBNE01AHLKH(1.5), FW1NBNE01A3PPX(2.5) |
|  | Ras GTP-binding nuclear protein | PUT-183a-Panax_ginseng-6102(2) |
|  | ATP synthase beta subunit | PUT-183a-Panax_ginseng-20342(3) |
|  | Methyltransferase | Contig312(3) |
|  | Unknown | PUT-183a-Panax_ginseng-8191(2), PUT-183a-Panax_ginseng-9869(2.5) |
| MIR1863 | Metalloproteinase | PUT-183a-Panax_ginseng-8137(2.5) |
|  | Metallothionein-like protein | PUT-183a-Panax_ginseng-17151(3), Contig979(3) |
|  | Polyubiquitin | Contig1203(3) |
| MIR2118 | TIR-NBS -LRR resistance protein | PUT-183a-Panax_ginseng-344(2.5) |
| MIR4376 | Ca^2+^-transporting ATPase | Contig2586(2) ^a^ |
|  | Coatomer subunit beta | Contig1190(3) |
|  | NADH dehydrogenase | PUT-183a-Panax_ginseng-6252(3) |
| MIR5072 | ZIP zinc transporter | PUT-183a-Panax_ginseng-14146(2.5) |
|  | H+-pyrophosphatase | Contig1376(3) |
| MIR5079 | Peroxidase | PUT-183a-Panax_ginseng-13580(3) |
|  | Unknown | PUT-183a-Panax_ginseng-2870(3), Contig2794(3) |
| MIR5139 | Exosome complex exonuclease RRP40 | FW1NBNE01A9X62(3) |

^a^ 5’RLM-RACE validated targets.
