# Supplementary material for: High-Throughput Sequencing and Characterization of the Small RNA Transcriptome Reveal Features of Novel and Conserved MicroRNAs in Panax ginseng
Source: PLoS One. 2012 Sep 4;7(9):e44385. doi: 10.1371/journal.pone.0044385 (PMC3433442; doi:10.1371/journal.pone.0044385)
Supplement: Table S5 — Sequencing analysis of miRNA amplicons from stem-loop RT-PCR. (DOC) [file pone.0044385.s007.doc]

**Table S5.** Sequencing analysis of miRNA amplicons from stem-loop RT-PCR.

| **miRNA name** | **miRNA sequence (5'→3')** | **Sequencing result ^a^** | **Ratio ^b^** |
| --- | --- | --- | --- |
| miR6135i | AATTGGCCAATAGAATACTGACAC | CGGC*AATTGGCCAATAGAATA***CTGACAC** | 5/5 |
| miR6135e2/j | AATTGACTAATAGAATACTGACAC | GCGGCGG*AATTGACTAATAGAAT***ACTGACAC** | 6/6 |
| miR6136b | TCATACAACCGTCGTCTATAC | GGCGG*TCATACAACCGTCGT***CTATAC** | 5/5 |
| miR6143b-3p | CAGCACTGTATTGAACATGAA | GCGGCGG*CAGCACTGTATTG***AACATGAA** | 5/5 |
| miR6139 | AAGAATCATTGGGAAGGGAAGAAA | CGG*AAGAATCATTGGGAAGGG***AAGAAA** | 5/5 |
| miR6140a | AATGTTTGTAGAATAGTTTGTGTC | CGGCGG*AATGTTTGTAGAATAGTT***TGTGTC** | 6/6 |
| miR6141 | TAACTAAATCTGGCCTGTAGCGGA | GGCGG*TAACTAAATCTGGCCT***GTAGCGGA** | 5/5 |

**^a^** The primer sequence is underlined. Partial miRNA sequence in the primers is shown in italic. miRNA sequence obtained by PCR is indicated in bold.

**^a^** The number of clones with integral 3’ ends of miRNAs / the number of total clones sequenced.
