# Supplementary material for: High-Throughput Sequencing and Characterization of the Small RNA Transcriptome Reveal Features of Novel and Conserved MicroRNAs in Panax ginseng
Source: PLoS One. 2012 Sep 4;7(9):e44385. doi: 10.1371/journal.pone.0044385 (PMC3433442; doi:10.1371/journal.pone.0044385)
Supplement: Table S6 — Primers used for 5′ RLM-RACE. (DOC) [file pone.0044385.s008.doc]

**Table S6.** Primers used for 5’ RLM-RACE.

| **Unigene ID** | **Gene-specific nesting primer** | **Gene-specific nested primer** |
| --- | --- | --- |
| FW1NBNE01AR7A8 | CATAGTAGAGCGTCTTAGGTCTTTTTA | GAGGAACGCTTACAAAGAAGCCCAAG |
| PUT-183a-Panax_ginseng-3299 | GCCAAGGCATGCATGTGGGCATTG | GTACTTCTGCCTGCCAACAGTCTGAT |
| PUT-183a-Panax_ginseng-12771 | GGAAGTGTACCTGGATATGCCAAGT | CCAAGTTCCCTGGAAATGGAGCAGAT |
| PUT-183a-Panax_ginseng-5279 | CGGGATTGGTGAGACATTGGAGAAG | CTAAAGAAGTAGAAGGCCGAGTTGATC |
| PUT-183a-Panax_ginseng-16749 | GGAGAACATCGGGATTGGTGAGAC | GAAGTAGAAGGCCGAGTTGATCCGGT |
| PUT-183a-Panax_ginseng-1543 | CAGCACCTATTGTAGTGACAGGATATG | GACTGATGTGGCATAGTGATAGTGCG |
| Contig1276 | TCGGGGTATAAGGTAGGCCAGGG | CCGTCTTATCCTTCGAATCAACCAGA |
| Contig2586 | GGTGTATCGAAATCGGCGAGTAGC | GCTTGCCTCCATTTTCTGAGTCGGTG |
| FW1NBNE01BIR6Z | AGGGGAAGGCTGCTCCATCTGCA | CCAACTGAATACAAGGGCAGGTTTTGG |
