# Supplementary material for: High-Throughput Sequencing and Characterization of the Small RNA Transcriptome Reveal Features of Novel and Conserved MicroRNAs in Panax ginseng
Source: PLoS One. 2012 Sep 4;7(9):e44385. doi: 10.1371/journal.pone.0044385 (PMC3433442; doi:10.1371/journal.pone.0044385)
Supplement: Table S7 — Primers used for poly(T) adaptor RT-PCR. (DOC) [file pone.0044385.s009.doc]

**Table S7.** Primers used for poly(T) adaptor RT-PCR.

| **Primer name** | **Sequence (5'-3')** |
| --- | --- |
| miR482a/b | TCTTGCCAATTCCTCCCATTCC |
| miR2118 | TTTCCTATTCCACCCATCCCAT |
| miR4376 | TGCAGGAGAGATGACGCCCATC |
| miR6135a/b | TGGTAAGTTGGTCAATTGGC |
| miR6135c/d/e.1 | GGGTAAGTTGGTCAATTGAC |
| miR6135f/g | GAGTAAGTTGGTCAATTGGC |
| miR6135h | GTAAGTTGGTCAATTGGC |
| miR6135i | AATTGGCCAATAGAATACTGACAC |
| miR6135e.2/j | AATTGACTAATAGAATACTGACAC |
| miR6135k | CGTGTCGATACTGTATTGGT |
| miR6136a.1 | TAGACGACGGTTGTATGACCG |
| miR6136a.2 | ACGGGTGAGTAAGATAAGGGGTAT |
| miR6136b | TCATACAACCGTCGTCTATAC |
| miR6137a/b | ATGAAAATTGTCGCTATAGATC |
| miR6138 | TACGTTTGGATTGAAGGAATGAAA |
| miR6139 | AAGAATCATTGGGAAGGGAAGAAA |
| miR6140a | AATGTTTGTAGAATAGTTTGTGTC |
| miR6140b | AATGTTTGTAGAATAATTTGTGTA |
| miR6140c | GCTGAGGTGGAGTATGCCACATC |
| miR6140d | CGTTGATGTGGCATACTTCACC |
| miR6141 | TAACTAAATCTGGCCTGTAGCGGA |
| miR6142 | GACGATTTTTTGGGCTATGACGAC |
| miR6143a | AGTACTGTATTGGGCATGAAG |
| miR6143b-5p | ACAATGTCGACACGCAGGCGGAGA |
| miR6143b-3p | CAGCACTGTATTGAACATGAA |
| Pg-5.8S | gtgaattgcagaatcccgtga |
| Reverse primer | GCGAGCACAGAATTAATACGAC |
