# Supplementary material for: High-Throughput Sequencing and Characterization of the Small RNA Transcriptome Reveal Features of Novel and Conserved MicroRNAs in Panax ginseng
Source: PLoS One. 2012 Sep 4;7(9):e44385. doi: 10.1371/journal.pone.0044385 (PMC3433442; doi:10.1371/journal.pone.0044385)
Supplement: Table S8 — Primers used for stem-loop RT-PCR. (DOC) [file pone.0044385.s010.doc]

**Table S8.** Primers used for stem-loop RT-PCR.

| **Primer name** | **Sequence (5'-3')** |
| --- | --- |
| miR6135i-RT | GTCGTATCCAGTGCAGGGTCCGAGGTATTCGCACTGGATACGACGTGTCA |
| miR6135i-F | CGGCAATTGGCCAATAGAATAC |
| miR6135j/e2-RT | GTCGTATCCAGTGCAGGGTCCGAGGTATTCGCACTGGATACGACGTGTCAGT |
| miR6135j/e2-F | GCGGCGGAATTGACTAATAGAAT |
| miR6135k-RT | GTCGTATCCAGTGCAGGGTCCGAGGTATTCGCACTGGATACGACACCAATAC |
| miR6135k-F | GCGGCGGCGTGTCGATACT |
| miR6136b-RT | GTCGTATCCAGTGCAGGGTCCGAGGTATTCGCACTGGATACGACGTATAG |
| miR6136b-F | GGCGGTCATACAACCGTCGT |
| miR6143b-3p-RT | GTCGTATCCAGTGCAGGGTCCGAGGTATTCGCACTGGATACGACTTCATGTT |
| miR6143b-3p-F | GCGGCGGCAGCACTGTATTG |
| miR6138-RT | GTCGTATCCAGTGCAGGGTCCGAGGTATTCGCACTGGATACGACTTTCAT |
| miR6138-F | GCGGTACGTTTGGATTGAAGG |
| miR6139-RT | GTCGTATCCAGTGCAGGGTCCGAGGTATTCGCACTGGATACGACTTTCTT |
| miR6139-F | CGGAAGAATCATTGGGAAGGG |
| miR6140a-RT | GTCGTATCCAGTGCAGGGTCCGAGGTATTCGCACTGGATACGACGACACA |
| miR6140a-F | CGGCGGAATGTTTGTAGAATAGTT |
| miR6140d-RT | GTCGTATCCAGTGCAGGGTCCGAGGTATTCGCACTGGATACGACGGTGAA |
| miR6140d-F | GCGGCGTTGATGTGGCATAC |
| miR6141-RT | GTCGTATCCAGTGCAGGGTCCGAGGTATTCGCACTGGATACGACTCCGCTAC |
| miR6141-F | GGCGGTAACTAAATCTGGCCT |
| Reverse primer | CAGTGCAGGGTCCGAGGTAT |
| 5.8S-F | CGATGAAGAACGTAGCGAAATGC |
| 5.8S-R | GCAACTTGCGTTCAAAGACTCGA |
